# Preliminary Assessment of the Anti-inflammatory Activity of New Structural Honokiol Analogs with a 4′-*O*-(2-Fluoroethyl) Moiety and the Potential of Their ^18^F-Labeled Derivatives for Neuroinflammation Imaging

**DOI:** 10.3390/molecules26216630

**Published:** 2021-11-01

**Authors:** Daria D. Vaulina, Kira I. Stosman, Konstantin V. Sivak, Andrey G. Aleksandrov, Nikolai B. Viktorov, Nikolay N. Kuzmich, Mariia M. Kiseleva, Olga F. Kuznetsova, Natalia A. Gomzina

**Affiliations:** 1 Laboratory of Radiochemistry, N.P. Bechtereva Institute of Human Brain, 197376 St. Petersburg, Russia; uplavice@gmail.com (D.D.V.); kuznets@ihb.spb.ru (O.F.K.); 2 Laboratory of Drug Safety, Smorodintsev Research Institute of Influenza WHO National Influenza Centre of Russia, 197376 St. Petersburg, Russia; kira.stosman@influenza.spb.ru (K.I.S.); kvsivak@gmail.com (K.V.S.); forphchemistry@gmail.com (A.G.A.); nnkuzmich@gmail.com (N.N.K.); 3 Department of Organic Chemistry, Faculty of Chemical and Biotechnologies, St. Petersburg State Technological Institute (Technical University), 190013 St. Petersburg, Russia; kolki@mail.ru; 4 World-Class Research Center “Digital Biodesign and Personalized Healthcare”, I.M. Sechenov First Moscow State Medical University, 119991 Moscow, Russia; 5 Centre de Recherche du Centre Hospitalier Universitaire de Québec, Université Laval, Québec, QC G1V 4G2, Canada; mariia.kiseleva.1@ulaval.ca

**Keywords:** structural analogs of honokiol, fluorine-18, neuroinflammation, radioligand, anti-inflammatory activity, cyclooxygenase-2 (COX-2)

## Abstract

Neolignans honokiol and 4′-*O*-methylhonokiol (MH) and their derivatives have pronounced anti-inflammatory activity, as evidenced by numerous pharmacological studies. Literature data suggested that cyclooxygenase type 2 (COX-2) may be a target for these compounds in vitro and in vivo. Recent studies of [^11^C]MPbP (*4′-[^11^C]methoxy-5-propyl-1,1′-biphenyl-2-ol*) biodistribution in LPS (lipopolysaccharide)-treated rats have confirmed the high potential of MH derivatives for imaging neuroinflammation. Here, we report the synthesis of four structural analogs of honokiol, of which *4′-(2-fluoroethoxy)-2-hydroxy-5-propyl-1, 1′-biphenyl* (**F-IV**) was selected for labeling with fluorine-18 (T_1/2_ = 109.8 min) due to its high anti-inflammatory activity confirmed by enzyme immunoassays (EIA) and neuromorphological studies. The high inhibitory potency of **F-IV** to COX-2 and its moderate lipophilicity and chemical stability are favorable factors for the preliminary evaluation of the radioligand **[^18^F]F-IV** in a rodent model of neuroinflammation. **[^18^F]F-IV** was prepared with good radiochemical yield and high molar activity and radiochemical purity by ^18^F-fluoroethylation of the precursor with Boc-protecting group (**15**) with [^18^F]2-fluoro-1-bromoethane ([^18^F]FEB). Ex vivo biodistribution studies revealed a small to moderate increase in radioligand uptake in the brain and peripheral organs of LPS-induced rats compared to control animals. Pretreatment with celecoxib resulted in significant blocking of radioactivity uptake in the brain (pons and medulla), heart, lungs, and kidneys, indicating that **[^18^F]F-IV** is likely to specifically bind to COX-2 in a rat model of neuroinflammation. However, in comparison with [^11^C]MPbP, the new radioligand showed decreased brain uptake in LPS rats and high retention in the blood pool, which apparently could be explained by its high plasma protein binding. We believe that the structure of **[^18^F]F-IV** can be optimized by replacing the substituents in the biphenyl core to eliminate these disadvantages and develop new radioligands for imaging activated microglia.

## 1. Introduction

Neuroinflammation is a complex inflammatory process within the CNS and occurs as a response to infection, protein aggregation, trauma, and ischemia [1]. Positron emission tomography (PET) is an in vivo molecular imaging technique that provides unique capabilities for detecting biochemical changes in activated CNS glia (microglia and astrocytes) to quantify neuroinflammation [2]. Cyclooxygenase (COX) enzymes catalyze prostaglandin synthesis and are involved in the activation of inflammatory pathways leading to the release of cytokines, chemokines, nitric oxide, and reactive oxygen species [3]. Both known isoforms, COX-1 and COX-2, are expressed in the brain. COX-1 is constitutively expressed in the CNS (as a “house-keeping” enzyme) and is not upregulated by inflammation, although some studies have shown that COX-1 may play a prominent role in neuroinflammation [4]. The inducible type of cyclooxygenase COX-2 is rapidly and dramatically overexpressed under inflammatory conditions and, therefore, is one of the most attractive molecular targets for PET imaging of neuroinflammation. When developing successful COX-2 radioligands, in addition to their high affinity and selectivity to COX-2, many other factors should be considered, such as penetration of the blood–brain barrier (BBB), chemical stability in plasma, suitable kinetic parameters, metabolic profile excluding the entry of metabolites into the brain, low nonspecific binding, and lipophilicity (LogD_7.4_) ranging from 2 to 4, etc. [5,6]. Selective COX-2 inhibitors (COXIBs) are the main class of anti-inflammatory drugs currently used clinically, a prominent representative of which is celecoxib. Several derivatives and structural analogues of celecoxib labeled with carbon-11 (T_1/2_ = 20.4 min) and fluorine-18 (T_1/2_ = 109.8 min) have been developed for COX-2 imaging by PET over the past decade [7]. Of these, [^11^C]TMI is the most promising radioligand for in vivo targeting of COX-2 in neuroinflammation, since it has demonstrated uptake even to constitutive expression of COX-2 in baboon brains [8]. Exploring a 2-(4-methylsulfonylphenyl) pyrimidine scaffold, radioligands [^11^C]MC1 [9] and [^18^F]FMTP [10] have recently demonstrated in vivo binding to COX-2 in the brain of LPS-injected primates and mice, respectively. [^11^C]MC1 also successfully visualized the upregulation of COX-2 in patients with rheumatoid arthritis in first-in-human studies [9].

An alternative to clinically used COXIBs could be structural analogs of natural phenolic compounds, which are attracting research attention for their anti-inflammatory capacity [11,12]. In addition, these compounds do not have the serious adverse effects commonly associated with celecoxib and its analogs. Several flavonoid and stilbene derivatives have been labeled with PET radionuclides to target amyloid plaques, tau protein, and alpha-synuclein aggregates in Alzheimer’s and Parkinson’s diseases, which are associated with chronic neuroinflammation [13,14].

**Scheme 1:**
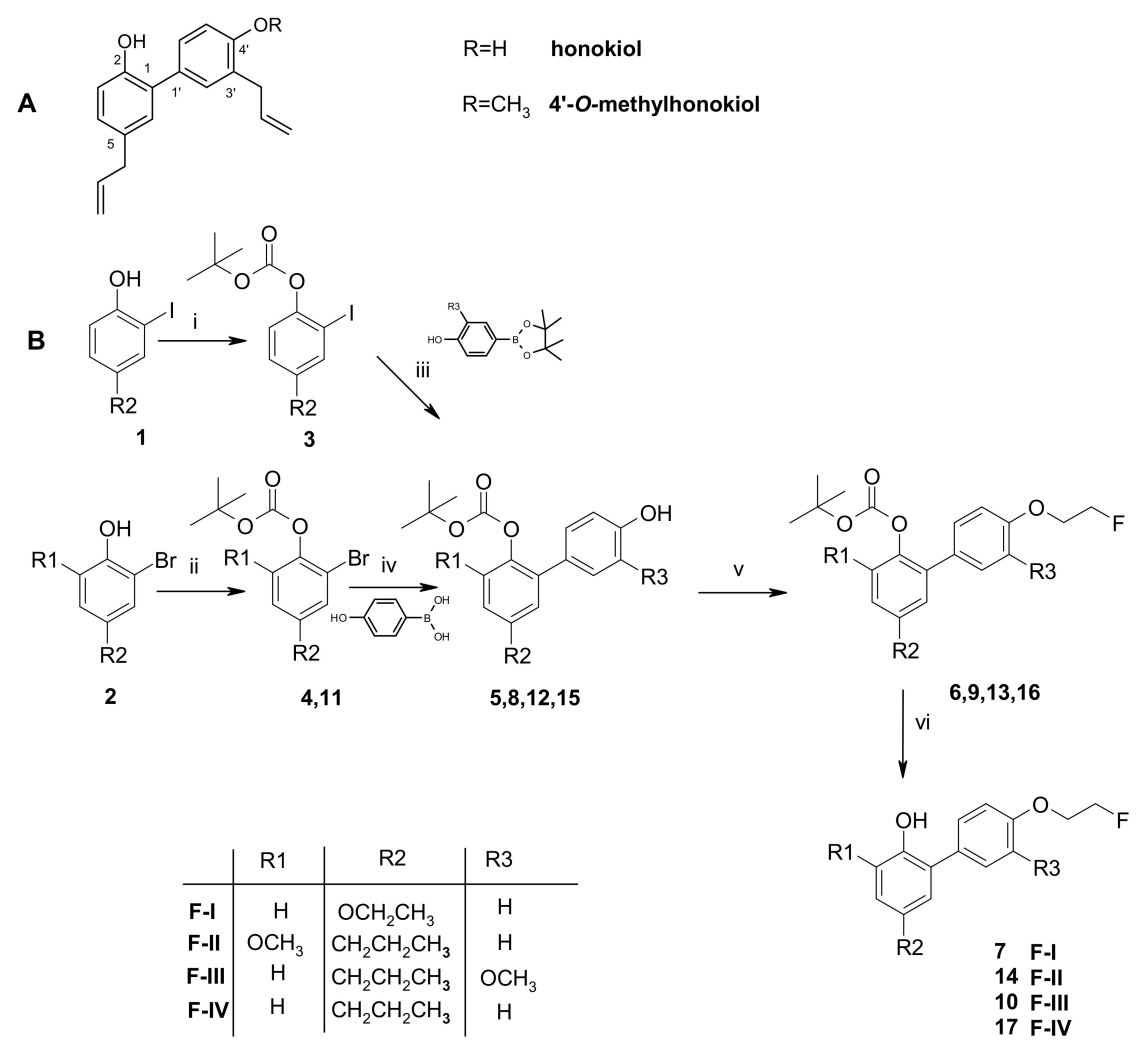
(**A**) Structures of neolignans honokiol and 4′-*O*-methylhonokiol (MH). (**B**) Syntheses of **F-I–F-IV** derivatives. Reagents and conditions: (i) and (ii)—Boc_2_O, DMAP, DCM, 20 °C, 17 h; (iii) and (iv)—Pd(Ph_3_P)_4_, Na_2_CO_3_, dioxane–water, 95 °C, 8 h; (v)—BrCH_2_CH_2_F, K_2_CO_3_, acetone, reflux, 24 h; (vi)—HCl conc., acetone, 20 °C, 16 h.

We recently proposed to explore the scaffold of 4′-*O*-methylhonokiol (MH in Scheme 1A), a natural biphenolic compound known for strong anti-inflammatory and neuroprotective activities [15,16,17], to design potential COX-2 radioligands. Radioligand [^11^C]MPbP (*4′-[^11^C]methoxy-5-propyl-1,1′-biphenyl-2-ol*) showed a high brain uptake in LPS (lipopolysaccharide)-treated rats compared to control animals [18]. These results inspired us to synthesize several new structural analogs of honokiol by replacing the methoxy group in MPbP with a fluoroethoxy group for convenient labeling with longer-lived fluorine-18 (**F-I–F-IV**, Scheme 1B). It is known that methoxy–fluoroethoxy substitution in aromatic rings gives a more stable compound against metabolic degradation than substitution with a fluoromethoxy moiety [19]. The ^18^F-labeled ligand has additional advantages in preclinical trials due to its 110 min half-life; in particular, it allows the study of biodistribution over a longer time to ensure accurate measurements and reduce the number of animals in experimental groups.

In this study, we aimed to produce an ^18^F-labeled analog of honokiol and to assess its potential for targeting COX-2 under inflammatory conditions. Based on the results of screening the anti-inflammatory activity of new structural analogs of honokiol with a 4′-*O*-(2-fluoroethyl) moiety (**F-I**–**F-IV**), we selected a candidate for radiolabeling. The initial evaluation of the radioligand **[^18^F]F-IV** was performed in a rat model of LPS-induced neuroinflammation.

## 2. Results and Discussion

### 2.1. Chemistry

Scheme 1B summarizes the chemistry used to prepare new honokiol structural analogs (**F-I–F-IV**)—potential anti-inflammation agents. The key step in the synthesis of these biphenyls was a Suzuki–Miyaura cross-coupling reaction of aryl iodides (**3**) or bromides (**4**, **11**) with aryl boronic compounds. The resulting intermediates (**5**, **8**, **12**, **15**) were converted into fluoroethoxy compounds (**6**, **9**, **13**, **16**), which were then deprotected with HCl acid.

### 2.2. Anti-Inflammatory Screening

**Figure 1:**
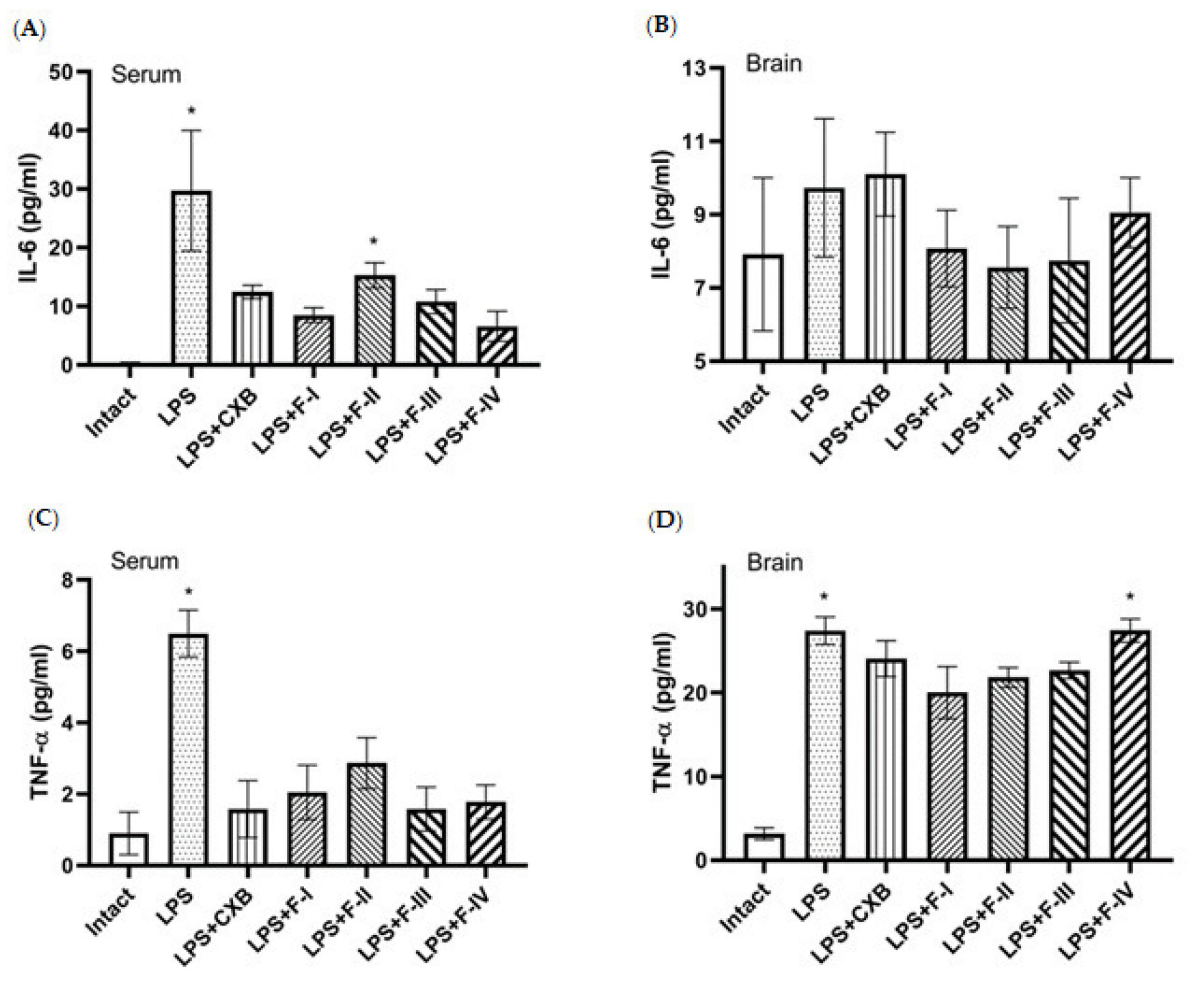
IL-6 and TNF-α levels 72 h after LPS injection in mice in serum (panels (**A**,**C**)) and in brain (panels (**B**,**D**)); * *p* < 0.05 statistically significant when compared with intact group; CXB—celecoxib; LPS—lipopolysaccharide.

**Figure 2:**
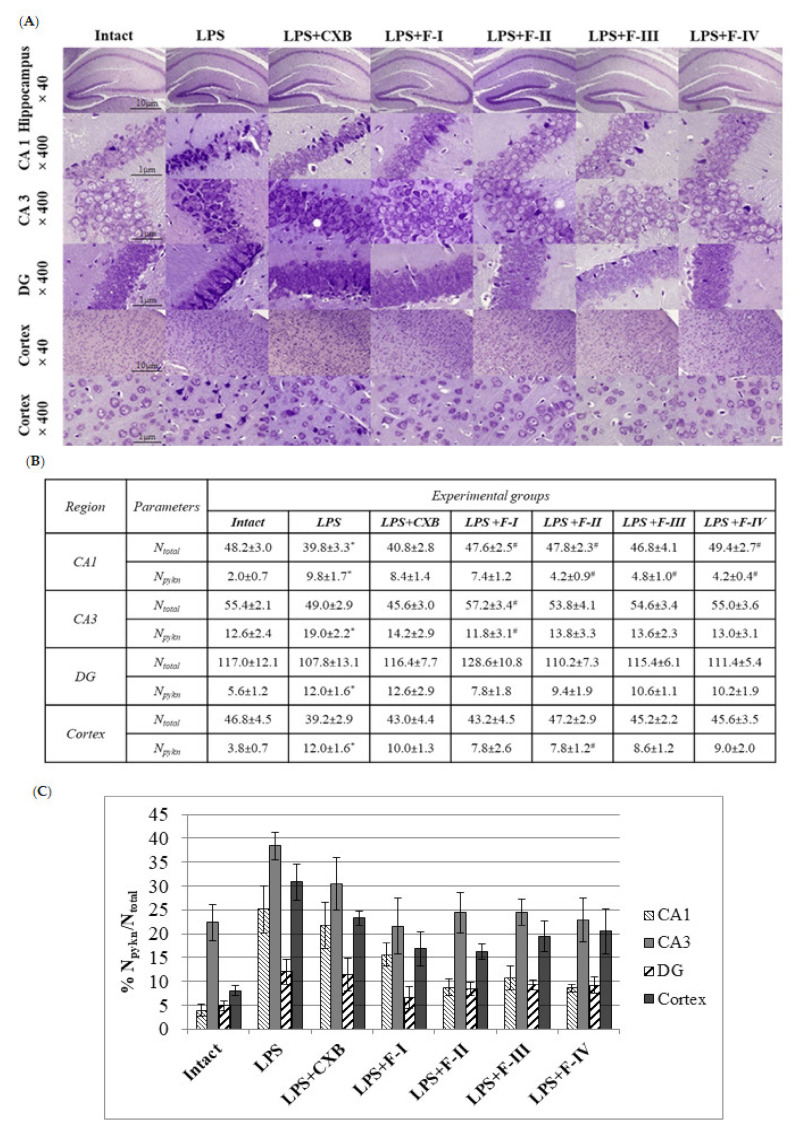
(**A**). Histological sections of the hippocampus (regions CA1, CA3, dentate gyrus DG) and posterior parietal cortex stained with Nissl cresyl violet. Effect of treatment with **F-I–F-IV** and celecoxib 72 h after LPS injection on linear density of neurons (N_total_)—panel (**B**) and on the number of pyknotic cells (N_pykn_)—panel (**C**). Data are provided as mean ± SEM (*n* = 3); *p* < 0.05—* significant when compared with the intact group, #—significant when compared with the LPS group.

**Figure 3:**
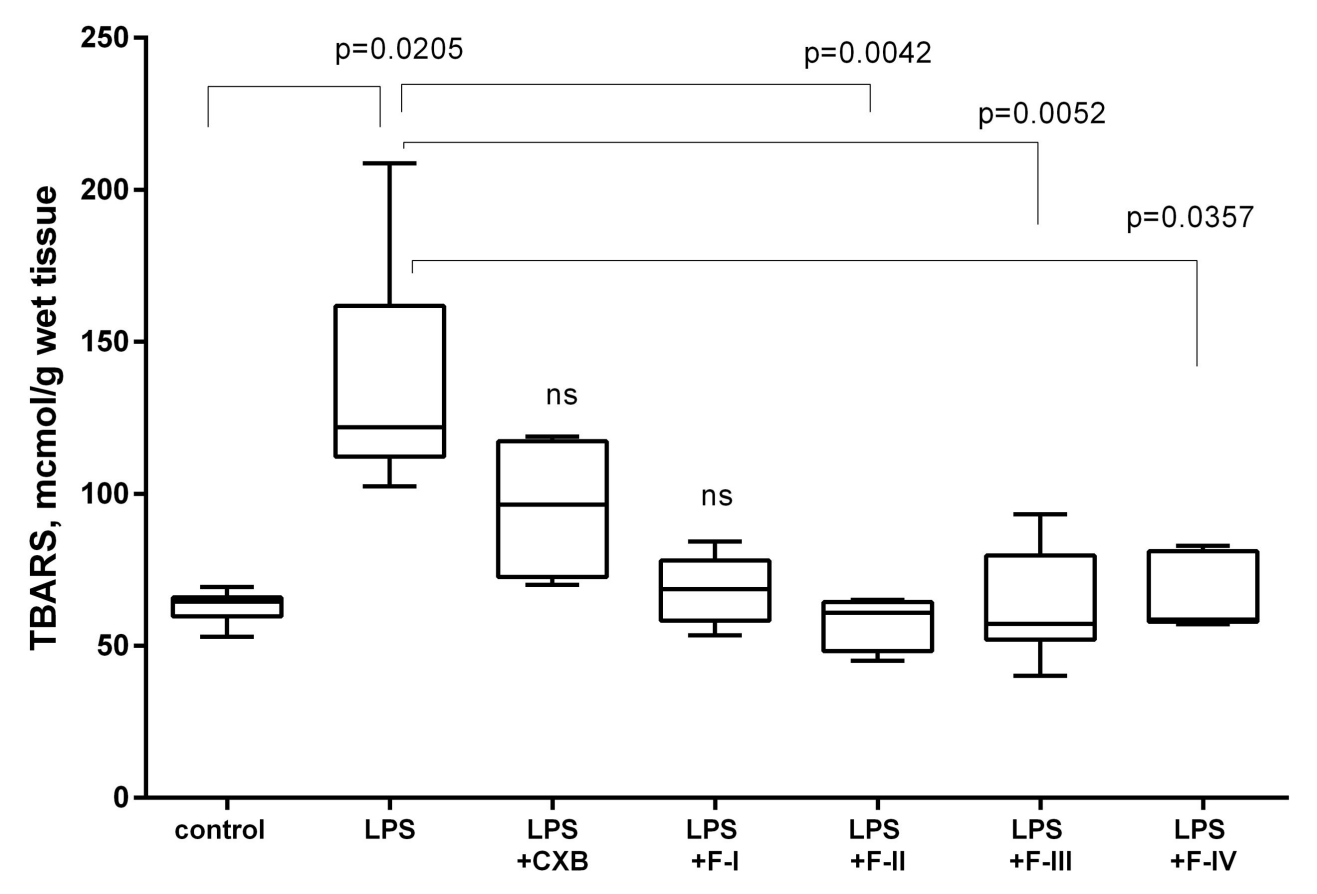
TBARS levels in mouse brain 72 h after LPS injection (*p*-value of significance, ns means non-significant).

It is known that peripheral inflammatory stimuli such as LPS, administered intraperitoneally, induce a profound immunological response in the brain, triggering microglia activation leading to neuroinflammation [20]. In our studies, LPS-induced neuroinflammation in mice was characterized by an increased expression of the pro-inflammatory cytokines IL-6 (interleukin-6) and TNF-α (tumor necrosis factor), a high level of lipid peroxide in the brain, as well as a significant increase in the number of pyknotic and damaged neurons in the hippocampus and cerebral cortex. These effects are shown in Figure 1, Figure 2 and Figure 3. The anti-inflammatory activity of the tested compounds **(F-I–F-IV),** manifested in a decrease in high levels of cytokines and lipid peroxides as well as in a decrease in neuronal pyknosis, was assessed using the reference drug celecoxib, a well-known NSAID and a selective COX-2 inhibitor.

Statistical analysis showed that LPS significantly increased serum levels of IL-6 and TNF-α, while treatment with compounds **F-I–F-IV** or celecoxib reduced them to levels seen in intact animals (Figure 1A,C). Moreover, **F-IV** exhibited an ability to inhibit serum cytokines comparable or even superior to that of celecoxib. In brain supernatants, the TNF-α level was significantly increased in all groups of LPS-induced mice compared to the minor level of the intact group. Although a moderate inhibition of TNF-α and IL-6 concentrations by **F-I–F-IV** compounds in the murine brain was found, it was not statistically significant (Figure 1B,D).

To assess the neuroprotective effect of the tested compounds, histological sections of four regions of the murine brain (CA1, CA3, DG of hippocampus and posterior parietal cortex) were analyzed. In morphometric studies, the total number of neurons (N_total_) and the number of pyknotic (degeneratively altered) neurons (N_pykn_) in the visual field were counted, as well as the ratio N_pykn_/N_total_. The results are shown in Figure 2A–C. The parietal cortex and hippocampus are among the most susceptible brain regions to induced neuroinflammation [21]. A decrease in the linear density of neurons (N_total_) by an average of 15–20% was observed in all selected regions of the brain in animals with LPS-induced neuroinflammation (LPS group, Figure 2B). This effect was accompanied by degenerative neuronal damage characterized by a decrease in cell size with changes in tinctorial properties of the cytoplasm and hypercondensation of nuclear chromatin. The number of pyknotic neurons (N_pykn_) in LPS-induced animals significantly exceeded the value in the intact mice in all studied brain regions: almost 5-fold in the C1 region, 1.5-fold in CA3, more than 2-fold in DG, and 3-fold in the parietal cortex (Figure 2B,C). The restoration of linear density and a decrease in the number of degenerative altered cells were observed in the brain of animals treated with the tested compounds. The greatest effects were noticed in the CA1 region for mice treated with **F-II**, **F-III**, and **F-IV**, and in CA3 for mice receiving **F-I**. In addition, the treatment with **F-II** resulted in a statistically significant decrease in the number of pyknotic neurons in the cortex. These data are likely to indicate the neuroprotective effect of the investigated compounds. Moreover, the observed therapeutic effect was slightly higher than that of the reference drug celecoxib.

It has been recognized that the involvement of lipid peroxidation as well as general oxidative stress is critical to the progression and regulation of inflammation, and lipid peroxides are key mediators of many neurodegenerative diseases [22,23]. The TBARS (Thiobarbituric Acid Reactive Species) assay has been used to measure lipid peroxidation products and to assess of the overall impact of oxidative stress agents such as LPS in animal models of neuroinflammation [24]. We hypothesized that the studied biphenyls, in addition to blocking neuroinflammation, can also suppress lipid peroxidation processes. As shown in Figure 3, the TBARS level in the brain of untreated LPS mice was 116% higher (*p* = 0.0205) compared to the control group.

The treatment of animals with the tested biphenyls reduced TBARS levels by 107% (**F-I**), 126% (**F-II**), 116% (**F-III**), and 111% (**F-IV**). Thus, **F-I–F-IV** biphenyls prevented the accumulation of lipid peroxidation products in the brain of LPS-injected mice. In contrast, treatment with celecoxib had little or no effect on lipid peroxide levels.

The obtained data indicate that our fluoroethoxy biphenyls not only have anti-inflammatory activity comparable or even superior to that of celecoxib, but also exhibit pronounced antioxidant properties. In our opinion, these biphenyls merit further investigation as potential anti-inflammatory agents in other animal models of neuroinflammation and peripheral inflammation.

Compound **F-IV** was selected from four candidates for radiolabeling because its structure is closest to the structure of MPbP, a previously studied MH derivative, the labeled analog of which showed high uptake in the brain of LPS-induced rats [18].

### 2.3. In Vitro COX-2 Inhibition

The anti-inflammatory activity exhibited by MH may result from the inhibition of molecular targets in activated microglia, such as COX-2 and cannabinoid receptor type 2 (CB-2) [25,26]. The **F-IV** inhibitory potency to human recombinant COX-1 and COX-2 was measured by enzymatic immunoassay (EIA) kit and the half-maximal inhibitor concentration (IC_50_) values were determined. **F-IV**, like MH, demonstrated good inhibitory potency against COX-2 at the submicromolar level: 0.09 and 0.06 μM, respectively (Table 1). At the same time, **F-IV** exhibited much lower selectivity for COX-2 (SI) than celecoxib (48 vs. >200), although slightly higher than MH and MPbP. Therefore, the question remains whether the activity of the tested biphenyl derivatives in suppressing neuroinflammation is directed only to COX-2 or to other molecular targets involved.

### 2.4. Computational Analysis

**Figure 4:**
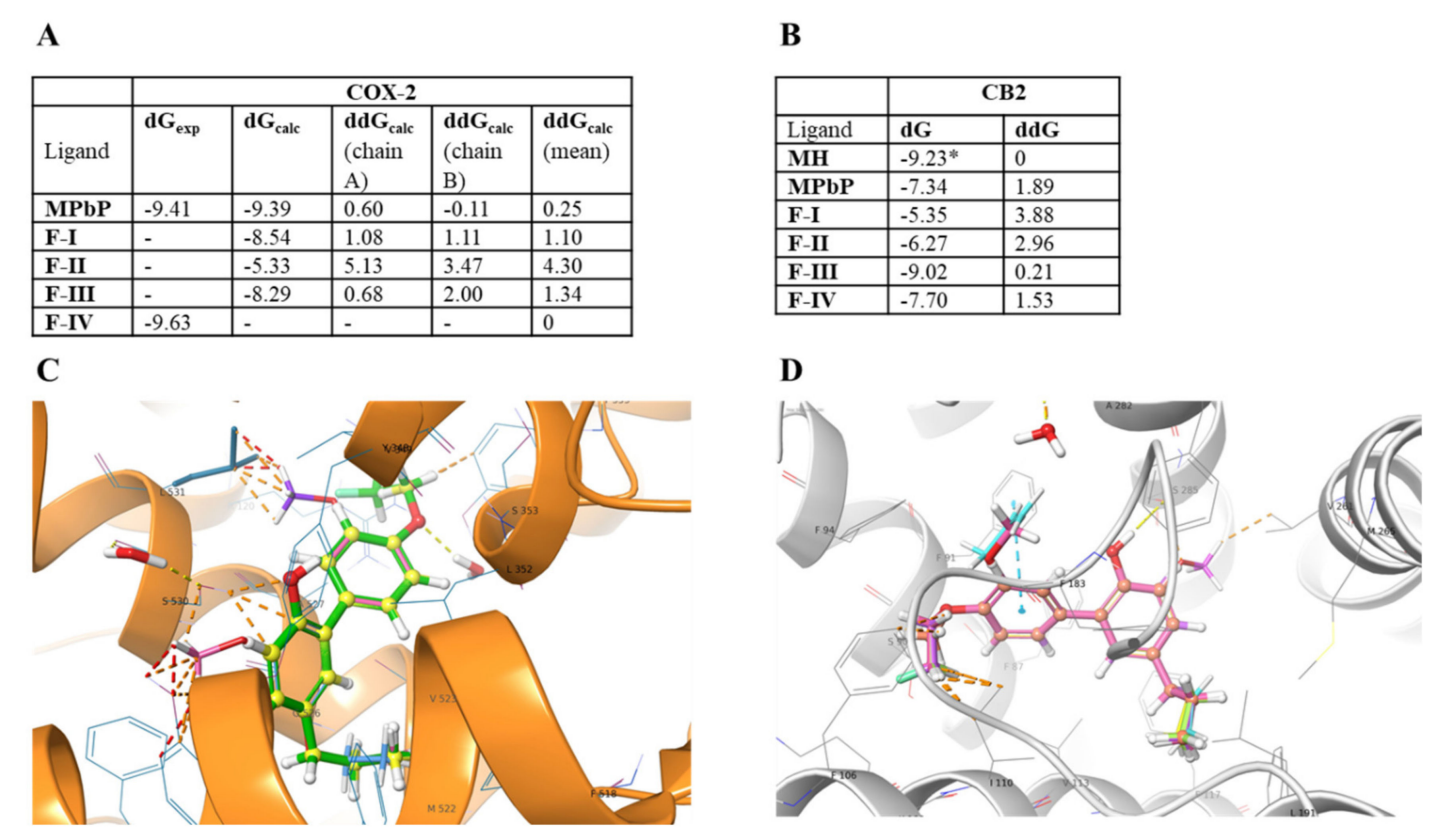
(**A**,**B**) panels—Computed relative binding free energies of **F-I**–**F-IV** to COX-2 (relative free energies (kcal/mol) are given in relation to **F-IV**) and to CB-2 (relative free energies are given in relation to MH, * from [26]), respectively. (**C**). Tested compounds aligned to **F-IV** by their maximum substructure at COX-2 binding site (MPbP—yellow, ball-and-stick; **F-I**—faded azure, thin tube; **F-II**—faded salmon, thin tube; **F-III**—violet, thin tube; **F-IV**—green, thick tube). (**D**). MH and tested compounds aligned to MH by their maximum substructure at CB-2 binding site (MH—cyan, thin tube; MPbP—faded red-orange, ball-and-stick; **F-I**—yellow, thin tube; **F-II**—magenta, thin tube; **F-III**—faded yellow-green, thick tube; **F-IV**—azure, thick tube).

We employed the alchemical free energy perturbation (FEP) method to predict the binding affinities of new compounds to COX-2 and CB-2 by calculating the relative free energy of the binding (ddG) of the ligands with these targets, and the results are given in Figure 4A,B. The complexes of CB-2 and COX-2 with **F-I–F-IV** are shown in Figure 4C, D. Calculated ddG between MPbP and **F-IV** for COX-2 was in excellent agreement with the experimental value (0.25 vs. 0.22 kcal/mol), confirming the correctness of the binding mode predicted. Both CB-2 and COX-2 have predominantly hydrophobic interactions with the tested ligands. In the case of CB-2, the ligands also have a hydrogen bond with S285 and p-p stacking interaction with F91. Analogously, COX-2 forms a bridge between S530 and OH-group of the ligands. Computed data support the preferential binding of MPbP and new fluoroethoxy biphenyls to COX-2 rather than to CB-2 in activated microglia except for the ligand **F-III**, which had an affinity for CB-2 very close to that of MH.

### 2.5. Radiochemistry

**Scheme 2:**
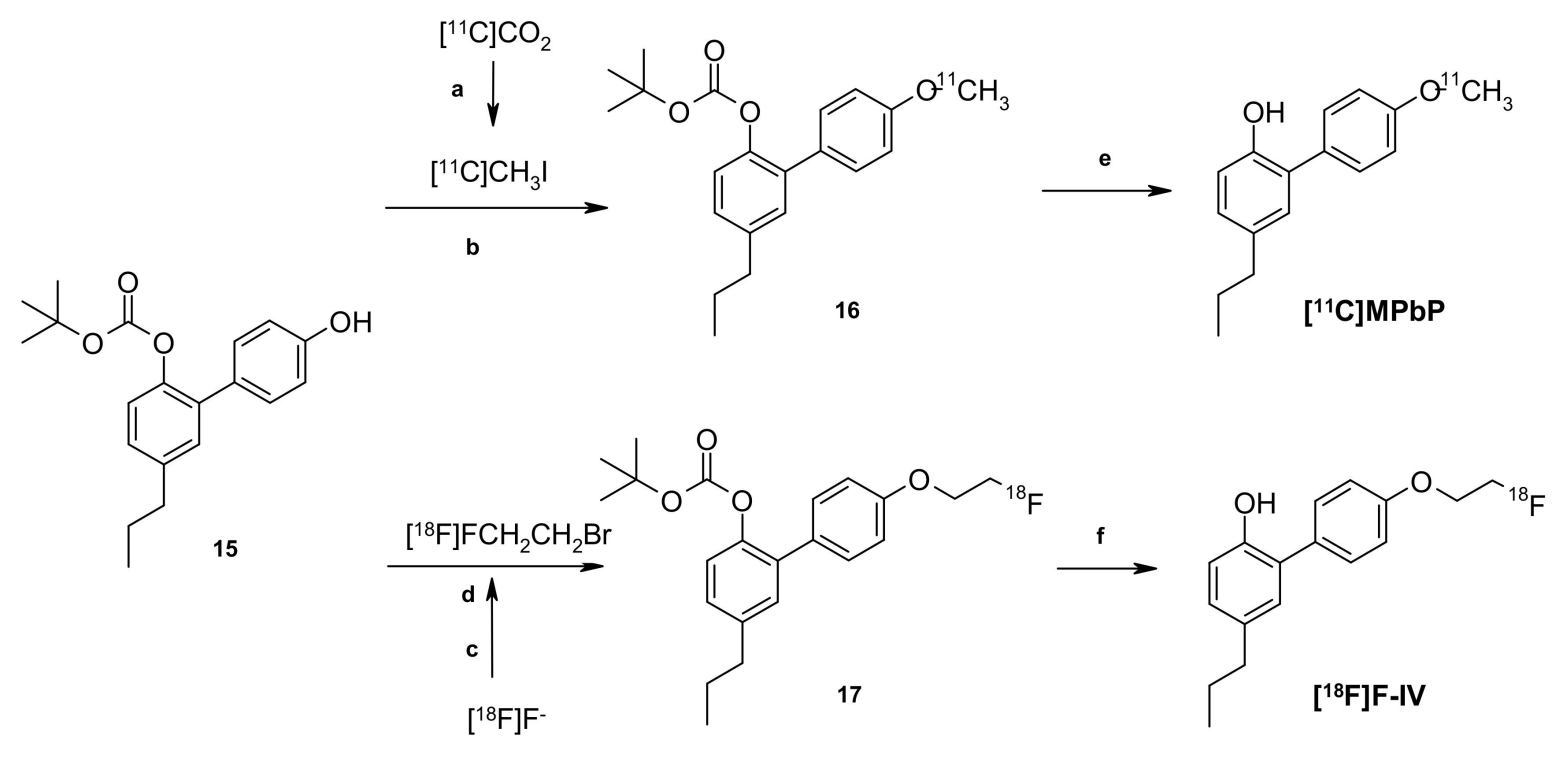
Radiosynthesis of **[^18^F]F-IV** and **[^11^C]MPbP.** Reagents and conditions: a—LiAlH_4_, 57% HI, 130 °C; b—NaOH, acetone, RT; c—[K^+^/K 2.2.2][^18^F^-^], BrCH_2_CH_2_OTs, *o*-DCB, 110 °C; d—KOMe, DMSO, 110 °C; e—HCl conc., 70 °C, 5 min; f—HCl conc., 100 °C, 10 min.

The radioligand **[^18^F]F-IV** (4′-*(2-[^18^F]fluoroethoxy)-2-hydroxy-5-propyl-1,1′-biphenyl*) was prepared like [^11^C]MPbP (*4′-[^11^C]methoxy-5-propyl-1,1′-biphenyl-2-ol*) from the labeling precursor **15** by a radioalkylation reaction (Scheme 2). To synthesize [^11^C]MPbP, [^11^C]iodomethane ([^11^C]CH_3_I) was used as the [^11^C]methylating agent [18]. **[^18^F]F-IV** was prepared using [^18^F]fluoroethylating agent [^18^F]2-fluoro-1-bromoethane ([^18^F]FEB) obtained from 2-bromoethyl tosylate and the [K^+^/K2.2.2][^18^F^−^] complex at 110 °C for 10 min [27]. Since [^18^F]FEB has high volatility (b.p. = 72 °C), it can be isolated from the reaction mixture by transfer with a carrier gas directly into the [^18^F]fluoroethylation vessel.

**Figure 5:**
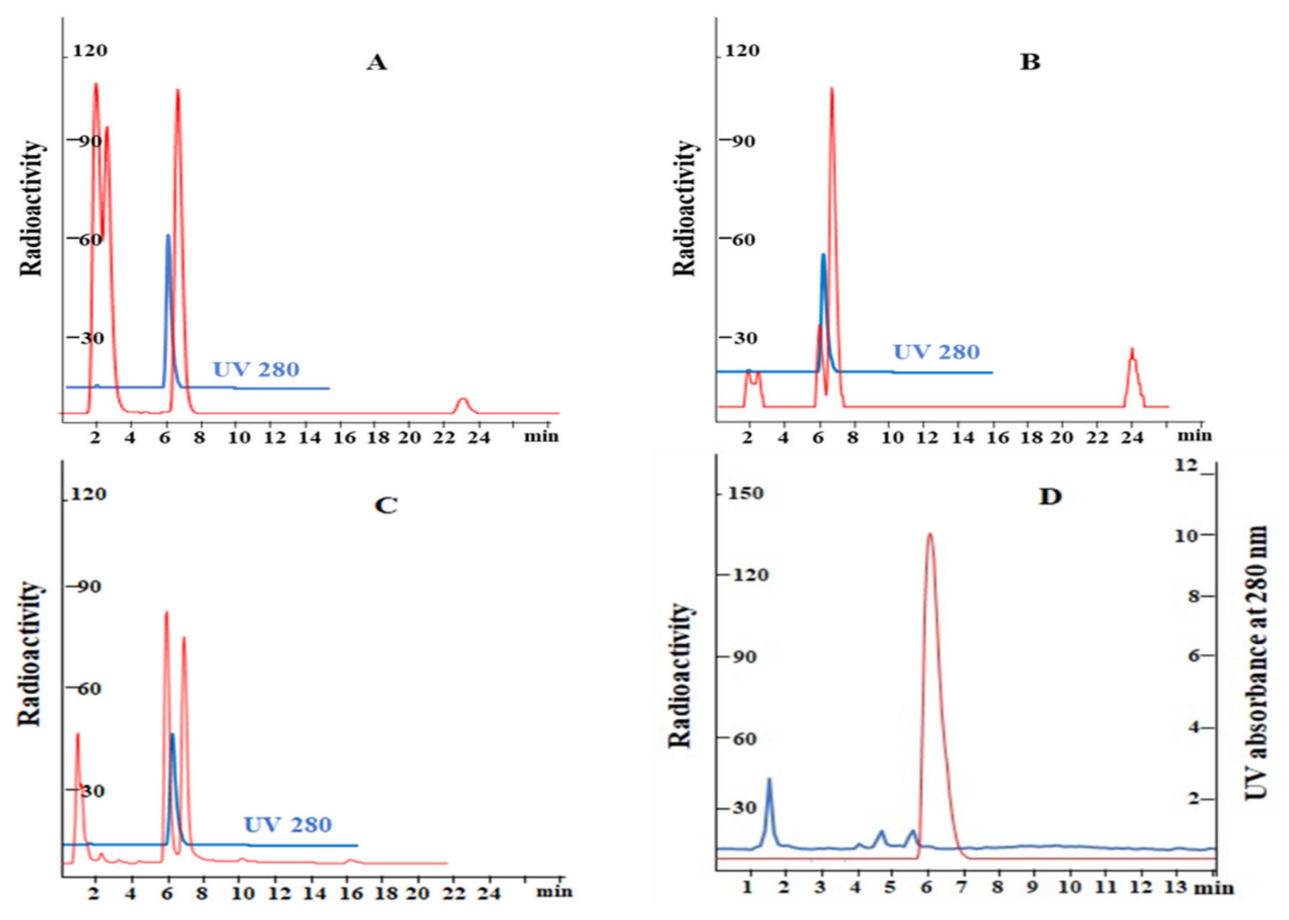
(**A**–**C**) panels—HPLC radiochromatograms of the crude reaction mixture after deprotecting of **17**, (**A**,**B**)—KOMe and NaOH, respectively, used as bases in [^18^F]fluoroethylation of **15**; (**C**)—NaOH used in [^18^F]fluoroethylation of 5-propyl-1,1′-biphenyl-2,4′-diol; blue line—UV chromatogram of unlabeled **F-IV** (reference compound). (**D**)—Chromatograms for HPLC analysis of the formulated radioligand **[^18^F]F-IV** (red line for radio- and blue line for UV-chromatograms).

We used potassium methoxide (KOMe) as a base in the [^18^F]fluoroethylation reaction, since in this case the conversion of intermediate **17** to **[^18^F]F-IV** by acid hydrolysis was quantitative (Figure 5A). Using NaOH or Bu_4_NOH, a radioactive by-product was obtained in which the [^18^F]fluoroethoxy group was in position 2 of the biphenyl structure and not in position 4′, as in **[^18^F]F-IV** (Figure 5B). The product and impurity are difficult to separate by preparative HPLC as they have close retention times. We assumed that the basic conditions of the radioalkylation reaction could facilitate the removal of the BOC protecting group from the precursor **15** [28] with the subsequent formation of biphenol having two equal positions (OH) for the attack of [^18^F]FEB. To confirm this hypothesis, a model reaction of [^18^F]FEB with 5-propyl-1,1′-biphenyl-2,4′-diol was carried out. The results of HPLC analysis indicated that the major reaction products were **[^18^F]F-IV** and by-product in a 45/55 ratio (Figure 5C). The cleavage of the BOC group from **15** by the action of NaOH or Bu_4_NOH is apparently catalyzed by a small amount of water [29] that is required for their dissolution in DMSO, while KOMe was dissolved directly in DMSO without the addition of water.

Semi-preparative HPLC provided **[^18^F]F-IV** with a radiochemical yield of 35% based on [^18^F]FEB radioactivity, and the molar activity was 35–40 GBq/μmol at the time of administration in rats.

The radiochemical purity of the formulated radioligand exceeded 97% and amounts of chemical impurities were negligible (<1µg/mL) (Figure 5D).

**[^18^F]F-IV** was radiochemically stable in human plasma for up to 60 min (92%), and no traces of [^18^F]defluorination were found, as measured by radio-HPLC. It is possible that the radioligand is metabolized through a different metabolic pathway.

The measured lipophilicity value (logD_7.4_) 3.30 ± 0.10 (*n* = 6) for **[^18^F]F-IV** was within the logD range (2–4) observed for most of the known PET radiotracers for brain imaging [6], and suggests that the radioligand is lipophilic enough to penetrate the BBB.

### 2.6. Ex Vivo Biodistribution Studies of [^18^F]F-IV

**Figure 6:**
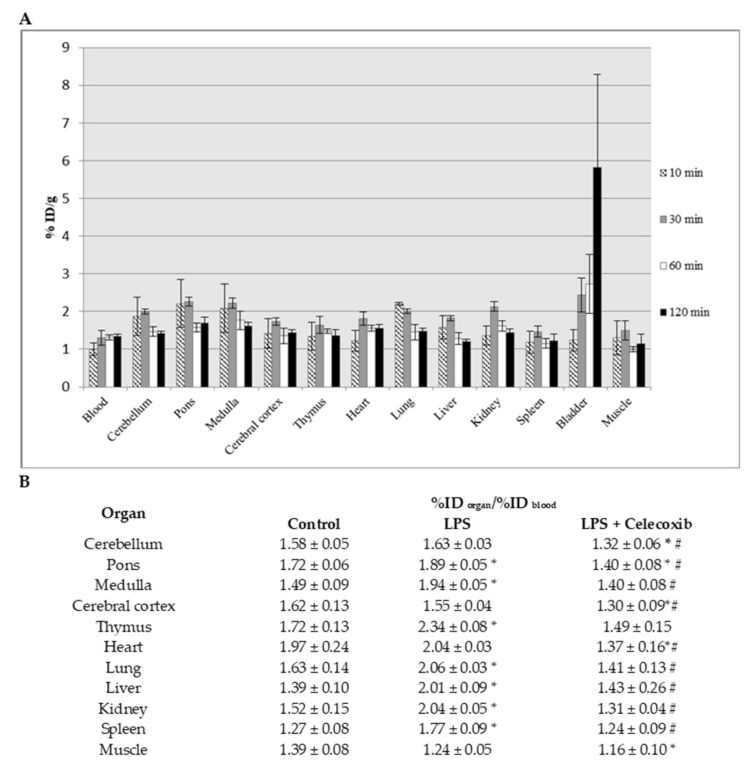
(**A**). Biodistribution data of **[^18^F]F-IV** in %ID/g (dose administered per gram of tissue) at 10 (*n* = 3), 30 (*n* = 4), 60 (*n* = 4), and 120 (*n* = 3) min post-injection in intact rats. (**B**). Biodistribution data of **[^18^F]F-IV** in %ID _organ_/%ID _blood_ at 60 min post-injection in LPS, LPS + celecoxib, and intact rats. Data are provided as mean ± SEM (*n* = 3) *p* < 0.05—* significant when compared with control group, # significant when compared with the LPS group.

Ex vivo biodistribution was intended as a preliminary assessment of the BBB permeability and overall distribution of **[^18^F]F-IV** in LPS-treated and intact Wistar rats. **[^18^F]F-IV** uptake by tissue was expressed as %ID/g (dose administered per gram of tissue). The obtained data in healthy animals showed a rapid distribution in most of the organs at 10 min. The radioligand seems to readily penetrate the BBB, as evidenced by the high initial rate of brain uptake with a peak value of 2.21 ± 0.64 and 2.09 ± 0.65 (%ID/g) in the pons and medulla, respectively, at 10 min post-injection, (Figure 6A). The ID data showed that the level of radioactivity remained high in both the brain and organs for more than 30 min post-injection, then gradually decreased and stabilized. The excretion pathway was through the kidneys, and 60 min after injection there was no obvious difference in **[^18^F]F-IV** uptake values between tissues. The lack of significant specific accumulation of radioactivity in the brain, heart, lungs, and kidneys most likely reflects the low baseline level of COX-2 expression in these organs in healthy animals [30]. It should be noted that relatively high initial levels of radioactivity in the blood pool persisted even 120 min after injection of **[^18^F]F-IV** (0.99 ± 0.17 vs. 1.35 ± 0.06%ID/g). This was the main difference in the distribution of **[^18^F]F-IV** and the previously reported [^11^C]MPbP in organs and tissues of intact rats. To describe **[^18^F]F-IV** accumulation in LPS-treated rats without or with celecoxib pretreatment (LPS block experiments), %ID/g values were corrected for radioactivity in the blood as %ID _organ_/%ID _blood_ for a more accurate assessment and exclusion of possible errors related to intravenously (i.v.) administration of the radioligand [31,32]. These results are depicted in Figure 6B. **[^18^F]F-IV** showed a small to moderate increase in brain uptake (5% in pons and 16% in medulla) in LPS-treated rats relative to saline-injected rats (control animals). A moderate increase in the radioactivity was observed in the peripheral organs of the LPS animals vs. controls: 12%, 16%, 20%, 20%, and 23% for lung, kidney, spleen, thymus, and liver, respectively. [^11^C]MPbP, in contrast to **[^18^F]F-IV**, showed a high uptake (up to 400%) in all examined organs and brain regions of LPS rats vs. healthy rats [18]. Blocking experiments with celecoxib were performed to clarify whether specific binding to COX-2 is responsible for the accumulation of radioligand in LPS rats. Pretreatment with celecoxib 30 min before the radioligand administration resulted in a reduced uptake of **[^18^F]F-IV** in almost all the extracted organs and tissues of LPS rats relative to background (control) levels.

The most significant blocking effect with a 20–32% reduction in radioactivity was observed in the brain (pons and medulla), heart, lung, and kidney, indicating that **[^18^F]F-IV** probably specifically binds to COX-2 in a rat model of LPS-induced neuroinflammation. Plasma protein binding (PPB) assays were performed to elucidate the reasons for the difference in pharmacokinetic profiles of the two radioligands.

### 2.7. Plasma Protein Binding (PPB) for [^18^F]F-IV and [^11^C]MPbP

After an injection is distributed in circulating blood, radioligands bind to plasma proteins and only rest unbound (free) fractions can join to the target of interest [33]. PPB in human and rat plasma samples was assessed by ultrafiltration, which is most commonly used in PET because of its relatively short analysis time and simplicity. The data in Table 2 revealed that both radioligands had a high PPB level, but **[^18^F]F-IV** is associated with proteins to a greater extent than [^11^C]MPbP: >98 vs. 92% for rats and >99 vs. 98% for humans, respectively. The measured high PPB of **[^18^F]F-IV** appears to be responsible for the high level of radioactivity in the blood, its retention in rat tissues, and for lower brain uptake (compared to MPbP) in LPS-treated rats, [34]. In this study, we did not evaluate the contribution of non-specific binding or off-target interactions of **[^18^F]F-IV** with proteins and phospholipids in the brain. However, they can also occur in the biodistribution of the radioligand **[^18^F]F-IV** due to its rather high lipophilicity and moderate selectivity for COX-2.

## 3. Experimental Procedure

### 3.1. General Chemistry 

All commercially available chemicals were used without any further purification. Melting points were determined by Kofler hot-stage (VEB Wägetechnik Rapido, PHMK 81/2969). Purifications by column chromatography on Merck silica gel 60 (0.035–0.070 mm) were performed. Analytical thin layer chromatography (TLC) on Merck 60 F254 silica gel plates with UV visualization (254 nm) was performed. NMR spectra were recorded on a Bruker Avance 400 spectrometer in CDCl_3_ (^1^H, ^13^C and ^19^F at 400.17, 100.62 and 376.54 MHz, respectively). HRMS (ESI) analysis was performed on a Bruker micrOTOF mass spectrometer. All manipulations of oxygen- and moisture-sensitive materials were conducted with a standard Schlenk technique.

### 3.2. Synthesis of Structural Analogs of Honokiol with a 4′-O-(2-Fluoroethyl) Moiety (**F-I –F-IV**)

The labeling precursor **15** *(4′-hydroxy-2-((1,1-dimethylethyloxycarbonyl)oxy)-5-propyl-1,1′-biphenyl-2-ol*) was obtained from **3** (*2-iodo-4-propylphenyl tert-butyl carbonate*) in a yield of 44%, and **F-IV** (*4′-(2-fluoroethoxy)-2-hydroxy-5-propyl-1,1′-biphenyl*) was obtained from **15** in a yield of 16% according to the previously described procedures [18,35].

#### 3.2.1. 5-Ethoxy-4′-(2-fluoroethoxy)-2-hydroxy-1,1′-biphenyl (**F-I**)

**F-I** was prepared in four steps from *2-bromo-4-ethoxyphenol* (**2**), which was obtained in 71% yield according to the procedure described in [36], (Figures S1 and S2).

*2-Bromo-4-ethoxyphenyl tert-butyl carbonate* (**4**): 4-Dimethylaminopyridine (0.01 g, 0.8 mmol) was added to stirring solution of (**2**) (1.65 g, 7.60 mmol) and Boc_2_O (1.75 g, 8.0 mmol) in 30 mL DCM. After 17 h at 20 °C, the solvent was removed at reduced pressure and the residue was dissolved in 12 mL hexane, filtered, and vacuum evaporated to afford (**4**) (2.30 g, yield 95%) as light-yellow oil. This product was used without further purification. ^1^H NMR (400 MHz; CDCl_3_) δ 1.41 (t, *J* = 7.2 Hz, 3H; CH_3_); 1.58 (s, 9H; t-Bu); 4.00 (q, *J* = 7.2 Hz, 2H; CH_2_O); 6.85 (dd, *J* = 8.8, 3.0 Hz, 1H, H-5); 7.11 (d, *J* = 8.8 Hz, 1H, H-6); 7.13 (d, *J* = 3.0 Hz, 1H, H-3), (Figure S3). ^13^C NMR (101 MHz; CDCl_3_) δ 14.8; 27.7 ((CH_3_)_3_); 64.3; 84.0; 114.7; 116.6; 118.7; 123.7; 142.1; 151.4; 157.3, (Figure S4). HRMS (ESI) *m*/*z*: calc. for C_13_H_17_BrO_4_: [M + Na]^+^ = 339.0202, found 339.0200; calc. [M + 2+Na]^+^ = 341.0183, found 341.0176.

*Tert-butyl 5-ethoxy-4′-hydroxy-1,1′-biphenyl-2-yl carbonate* (**5**): Pd(Ph_3_P)_4_ (0.15 g, 0.13 mmol) was added to the mixture of (**4**) (0.80 g, 2.52 mmol), 4-hydroxyphenylboronic acid (0.41 g, 3.0 mmol), aq. sodium carbonate 1M (11 mL, 11.0 mmol), and 1,4-dioxane (10 mL) under argon and the resulting mixture was stirred at 95 °C for 8 h. After cooling to RT, water (40 mL) was added and mixture was extracted with ethyl acetate 3 × 30 mL. The organic phase was dried over Na_2_SO_4_ and concentrated under reduced pressure. The residue (0.95 g) was purified by silica gel column chromatography (heptane/ethyl acetate = 5:1) to afford **5** (0.20, yield 24%) as light-yellow oil. ^1^H NMR (400 MHz, CDCl_3_) δ 1.35 (s, 9H, t-BuO); 1.43 (t, *J* = 7.2 Hz, 3H, CH_3_); 4.06 (q, *J* = 7.2 Hz, 2H, CH_2_O); 5.5–6.7 (brs, 1H, OH); 6.82 (d, *J* = 8.8 Hz, 2H); 6.85 (dd, *J* = 8.8, 2.8 Hz, 1H, H-4); 6.91 (d, *J* = 2.8 Hz, 1H, H-6); 7.10 (d, *J* = 8.8 Hz, 1H, H-3); 7.32 (d, *J* = 8.8 Hz, 2H), (Figure S5). ^13^C NMR (101 MHz, CDCl_3_) δ 14.9; 27.5 ((CH_3_)_3_); 64.0; 83.5; 113.7; 115.4; 116.4; 123.1; 129.5; 130.2; 135.6; 141.8; 152.2; 155.6; 156.9, (Figure S6). HRMS (ESI) ***m***/*z*: calc. for C_19_H_22_O_5_: [M + Na]^+^ = 353.1359, found 353.1343.

*Tert-butyl 5-ethoxy-4′-(2-fluoroethoxy)-1,1′-biphenyl-2-yl carbonate* (**6**): The mixture of (**5**) (0.10 g, 0.30 mmol), 1-bromo-2-fluoroethane (0.30 g, 2.36 mmol), K_2_CO_3_ (0.25 g, 1.8 mmol), and dry acetone (8 mL) was refluxed during 24 h. After filtering, the solid was washed with 15 mL acetone. The solution was concentrated under reduced pressure and the residue oil (0.15 g) was purified by column chromatography on silica gel (heptane/ ethyl acetate = 10/1) to afford **6** (0.098 g, 87% yield) as clear oil. ^1^H NMR (400 MHz, CDCl_3_) δ 1.36 (s, 9H; t-BuO); 1.44 (t, *J* = 7.2 Hz, 3H; CH_3_); 4.07 (q, *J* = 7.2 Hz, 2H; CH_2_O); 4.26 (dm, *J*^3^_HF_ = 28 Hz, 2H, CH_2_O); 4.79 (dm, *J*^2^_HF_ = 47 Hz, 2H, CH_2_F); 6.87 (dd, *J* = 8.8, 3.0 Hz, 1H, H-4); 6.92 (d, *J* = 3.0 Hz, 1H, H-6); 6.99 (d, *J* = 8.8 Hz, 2H); 7.11 (d, *J* = 8.8 Hz, 1H, H-3); 7.43 (d, *J* = 8.8Hz, 2H), (Figure S7). ^13^C NMR (101 MHz, CDCl_3_) δ 15.0; 27.5 ((CH_3_)_3_); 64.0; 67.3 (d, *J*^2^_CF_ = 20 Hz, OCH2); 82.0 (d, *J*^1^_CF_ = 170 Hz, FCH2); 83.1; 113.8; 114.6; 116.4; 123.2; 130.2; 130.7; 135.4; 141.8; 152.0; 156.9; 158.0, (Figure S8). ^19^F NMR (376.5 MHz, CDCl_3_) δ -223.8, (Figure S9). HRMS (ESI): mass calculated for C_21_H_25_FO_5_: [M + Na]^+^ = 399.1578; found 399.1564.

*5-Ethoxy-4′-(2-fluoroethoxy)-2-hydroxy-1,1′-biphenyl* (**7**), (**F**-**I**): The mixture of **6** (0.10 g, 0.26 mmol), 0.5 mL HCl conc. (12N), and acetone (2 mL) was stirred for 16 h at 20 °C. After evaporation at reduced pressure, the residue was purified by column chromatography on silica gel (heptane/DCM = 4/1) to afford **7** (0.05 g, 67% yield) as white solid, m.p. 88–89 °C (hexane/diethyl ether 1/1). ^1^H NMR (400 MHz, CDCl_3_) δ 1.42 (t, *J* = 7.2 Hz, 3H, CH_3_); 4.03 (q, *J* = 7.2 Hz, 2H; CH_2_O); 4.28 (dm, *J*^3^_HF_ =28 Hz, 2H, CH_2_O); 4.40–4.60 (brs, 1H, OH); 4.80 (dm, *J*^2^_HF_ =47 Hz, 2H, CH_2_F); 6.77–6.95 (m, 3H, H-3, H-4, H-6); 7.06 (d, *J* = 8.8 Hz, 2H); 7.43 (d, *J* = 8.8 Hz, 2H), (Figure S10). ^13^C NMR (101 MHz, CDCl_3_) δ 15.0; 64.2; 67.2 (d, *J*^2^_CF_ = 20 Hz, OCH2); 81.0 (d, *J*^1^_CF_ = 170 Hz, FCH2); 114.9; 115.3; 116.2; 116.5; 128.3; 130.1; 130.3; 146.5; 152.9; 158.1, (Figure S11). ^19^F NMR (376.5 MHz, CDCl_3_) δ -223.7, (Figure S12). HRMS (ESI): mass calculated for C_16_H_17_FO_3_ [M + Na]^+^ = 299.1054; found 299.1060. 

#### 3.2.2. 4′-(2-Fluoroethoxy)-3′-methoxy-5-propyl-2-hydroxy-1,1′-biphenyl (**F-III**) 

*Tert-butyl 4′-hydroxy-3′-methoxy-5-propyl-1,1′-biphenyl-2-yl carbonate* (**8**): This compound was prepared by the same procedure as described for **5** using 2-iodo-4-propylphenyl tert-butyl carbonate **3** [35] and 2-methoxy-4-(4,4,5,5-tetramethyl-1,3,2-dioxaborolan-2-yl)phenol. The yield of light-yellow oil was 46%. ^1^H NMR (400 MHz, CDCl_3_) δ 0.99 (t, *J* = 7.2 Hz, 3H, CH_3_); 1.35 (s, 9H; t-BuO); 1.68 (m, 2H; CH_2_); 2.63 (m, 2H, CH_2_Ar); 3.95 (s, 3H, OCH_3_); 5.65 (brs, 1H, OH); 6.99 (m, 2H); 7.03 (d, *J* = 1.6 Hz, 1H); 7.10 (d, *J* = 8.0 Hz, 1H); 7.15 (dd, *J* = 8.4, 2.0 Hz, 1H); 7.22 (d, *J* = 2.0 Hz, 1H), (Figure S13). ^13^C NMR (101 MHz, CDCl_3_) δ 14.0; 24.7; 27.5 ((CH_3_)_3_); 37.6; 56.1; 83.1; 111.7; 114.4; 122.2 (two C); 128.2; 129.8; 130.7; 134.3; 140.9; 145.2; 146.2; 146.3; 151.8, (Figure S14). HRMS (ESI): mass calculated for C_21_H_26_O_5_ [M-H]^-^ = 357.1697; found 357.1688.

*Tert-butyl 4′-(2-fluoroethoxy)-3′-methoxy-5-propyl-1,1′-biphenyl-2-yl carbonate* (**9**). This compound was prepared from **8** by the same procedure as described for **6**. The yield of white solid was 88% with m.p. 57–58 °C (hexane). ^1^H NMR (400 MHz, CDCl_3_) δ 0.99 (t, *J* = 7.2 Hz, 3H, CH_3_); 1.35 (s, 9H; t-BuO); 1.69 (m, 2H, CH_2_); 2.64 (m, 2H, CH_2_Ar); 3.93 (s, 3H, OCH_3_); 4.33 (dm, *J*^3^_HF_ = 28 Hz, 2H, CH_2_O); 4.82 (dm, *J*^2^_HF_ = 47 Hz, 2H, CH_2_F); 6.98 (d, *J* = 8.0 Hz, 1H); 7.02 (dd, *J* = 8.0, 2.0 Hz, 1H); 7.08 (d, *J* = 1.6 Hz, 1H); 7.10 (d, *J* = 8.0 Hz, 1H); 7.16 (dd, *J* = 8.0, 2.0 Hz, 1H); 7.23 (d, *J* = 2.0 Hz, 1H), (Figure S15). ^13^C NMR (101 MHz, CDCl_3_) δ 14.0; 24.7; 27.5 ((CH_3_)_3_); 37.6; 56.1; 68.7 (d, *J*^2^_CF_ = 21 Hz, OCH2); 82.1 (d, *J*^1^_CF_ = 171 Hz, FCH2); 83.1; 113.0; 114.5; 121.4; 122.2; 128.4; 130.7; 131.8; 134.1; 140.9; 146.2; 147.3; 149.5; 151.7, (Figure S16). ^19^F NMR (376.5 MHz, CDCl_3_) δ -223.5, (Figure S17). HRMS (ESI): mass calculated for C_23_H_29_FO_5_ [M + Na] ^+^ = 427.1891; found 427.1884.

*4′-(2-fluoroethoxy)-3′-methoxy-5-propyl-2-hydroxy-1,1′-biphenyl (**10**),* **F-III.**

This compound was prepared from **9** by the same procedure as described for **7**. The yield of clear oil was 83%. ^1^H NMR (400 MHz, CDCl_3_) δ 0.98 (t, *J* = 7.2 Hz, 3H, CH_3_); 1.66 (m, 2H, CH_2_); 2.58 (m, 2H, CH_2_Ar); 3.93 (s, 3H, OCH_3_); 4.36 (dm, *J*^3^_HF_ = 27 Hz, 2H, CH_2_O); 4.83 (dm, *J*^2^_HF_ = 47 Hz, 2H, CH_2_F); 5.17 (brs, 1H, OH); 6.92 (d, *J* = 8.0 Hz, 1H); 7.00–7.12 (m, 5H), (Figure S18). ^13^C NMR (101 MHz, CDCl_3_) δ 14.0; 24.9; 37.3; 56.2; 68.7 (d, *J*^2^_CF_ = 21 Hz, OCH2); 82.1 (d, *J*^1^_CF_ = 171 Hz, FCH2); 113.1; 114.8; 115.6; 121.3; 127.6; 129.0; 130.1; 131.2; 135.1; 147.7; 150.4; 150.5, (Figure S19). ^19^F NMR (376.5 MHz, CDCl_3_) δ -223.4, (Figure S20). HRMS (ESI): mass calculated for C_18_H_21_FO_3_ [M-H ]^-^ = 303.1391; found 303.1385.

#### 3.2.3. 4′-(2-Fluoroethoxy)-3-methoxy-5-propyl-2-hydroxy-1,1′-biphenyl (**F-II**)

*2-Bromo-6-methoxy-4-propylphenyl tert-butyl carbonate* (**11**): This compound was prepared from 2-bromo-6-methoxy-4-propylphenol (**2**) by the same procedure as described for **4**. The yield of compound **2** obtained according to [37] was about 74%. Product **11** obtained as clear oil (2.65 g, yield 95%) was used without further purification.^1^H NMR (400 MHz; CDCl_3_) δ 1.01 (t, *J* = 7.2 Hz, 3H, CH_3_); 1.57 (s, 9H, t-Bu); 1.65 (m, 2H, CH_2_); 2.68 (m, 2H, CH_2_Ar); 3.65 (s, 3H, OCH_3_); 6.82 (s, 1H, H-5); 7.30 (s, 1H, H-3), (Figure S21). ^13^C NMR (101 MHz; CDCl_3_) δ 14.0; 23.3; 27.7 ((CH_3_)_3_); 38.3; 56.2; 83.8; 113.6; 114.0 (C-5); 126.4 (C-3); 138.6; 140.4; 150.5; 151.4, (Figure S22). HRMS (ESI) *m*/*z*: calc. for C_15_H_21_BrO_4_: [M + Na]^+^ = 367.0515, found 367.0512; calc. [M + 2+Na]^+^ = 369.0495, found 369.0489.

*Tert-butyl 4′-hydroxy-3-methoxy-5-propyl-1,1′-biphenyl-2-yl carbonate* (**12**): This compound was prepared by the same procedure as described for **5** using **11** and 4-hydroxyphenylboronic acid. The yield was 21%, clear syrup. ^1^H NMR (400 MHz, CDCl_3_) δ 0.85 (t, *J* = 7.2 Hz, 3H, CH_3_); 1.51 (m, 2H, CH_2_); 1.57 (s, 9H, t-Bu); 2.53 (m, 2H, CH_2_Ar); 3.90 (s, 3H, OCH_3_); 4.40–5.30 (brs, 1H, OH); 6.81 (d, *J* = 8.4 Hz, 2H); 6.86 (s, 1H); 6.96 (s, 1H); 7.16 (d, *J* = 8.4 Hz, 2H), (Figure S23). ^13^C NMR (101 MHz, CDCl_3_) δ 14.2; 24.7; 27.8 ((CH_3_)_3_); 35.3; 56.2; 83.7; 113.3; 115.0; 124.1; 130.7; 133.3; 134.1; 137.6; 139.2; 149.9; 152.2; 154.7, (Figure S24). HRMS (ESI): mass calculated for C_21_H_26_O_5_ [M + Na]^+^ = 381.1672; found 381.1664.

*Tert-butyl 4′-(2-fluoroethoxy)-3-methoxy-5-propyl-1,1′-biphenyl-2-yl carbonate* (**13**). This compound was prepared from **12** by the same procedure as described for **6**. The yield was 82%, white solid, m.p. 45–46 °C (hexane). ^1^H NMR (400 MHz, CDCl_3_) δ 0.85 (t, *J* = 7.2 Hz, 3H, CH_3_); 1.51 (m, 2H, CH_2_); 1.57 (s, 9H, t-BuO); 2.53 (m, 2H, CH_2_Ar); 3.90 (s, 3H, OCH_3_); 4.28 (dm, *J*^3^_HF_ = 28 Hz, 2H, CH_2_O); 4.81 (dm, *J*^2^_HF_ = 47 Hz, 2H, CH_2_F); 6.87 (s, 1H); 6.96 (d, *J* = 8.8 Hz, 2H); 6.97 (s, 1H); 7.22 (d, *J* = 8.8 Hz, 2H), (Figure S25). ^13^C NMR (101 MHz, CDCl_3_) δ 14.2; 24.7; 27.8 ((CH_3_)_3_); 35.3; 56.2; 67.2 (d, *J*^2^_CF_ = 20 Hz, OCH2); 82.1 (d, *J*^1^_CF_ = 170 Hz, FCH2); 83.4; 113.3; 114.2; 124.0; 130.7; 133.9; 134.1; 137.7; 139.1; 150.1; 151.9; 157.4, (Figure S26). ^19^F NMR (376.5 MHz, CDCl_3_) δ -223.8, (Figure S27). HRMS (ESI): mass calculated for C_23_H_29_FO_5_ [M + Na]^+^ = 427.1891; found 427.1904.

*4′-(2-Fluoroethoxy)-3-methoxy-5-propyl-2-hydroxy-1,1′-biphenyl* (**14**), **F**-**II.** This compound was prepared from **13** by the same procedure as described for **7**. The yield was 73% (clear oil). ^1^H NMR (400 MHz, CDCl_3_) δ 0.85 (t, *J* = 7.2 Hz, 3H, CH_3_); 1.50 (m, 2H, CH_2_); 2.51 (m, 2H, CH_2_Ar); 3.95 (s, 3H, OCH_3_); 4.29 (dm, *J*^3^_HF_ = 28 Hz, 2H, CH_2_O); 4.83 (dm, *J*^2^_HF_ = 48 Hz, 2H, CH_2_F); 5.54 (brs, 1H, OH); 6.79 (s, 1H); 6.81 (s, 1H); 6.97 (d, *J* = 8.2 Hz, 2H); 7.23 (d, *J* = 8.2 Hz, 2H), (Figure S28). ^13^C NMR (101 MHz, CDCl_3_) δ 14.1; 25.0; 35.0; 56.1; 67.2 (d, *J*^2^_CF_ = 20 Hz, OCH2); 82.1 (d, *J*^1^_CF_ = 170 Hz, FCH2); 111.6; 114.2; 116.3; 130.6; 132.2; 134.4; 134.9; 143.2; 145.7; 157.2, (Figure S29). ^19^F NMR (376.5 MHz, CDCl_3_) δ −223.7, (Figure S30). HRMS (ESI): mass calculated for C_18_H_21_FO_3_ [M + Na]^+^ = 327.1367; found 327.1368.

#### 3.2.4. 5-Propyl-1,1′-biphenyl-2,4′-diol (Model Compound)

In argon atmosphere, Pd(Ph_3_P)_4_ (0.08 g, 0.07 mmol) was added to the mixture of 2-iodo-1-((1,1-dimethylethoxycarbonyl)oxy)-4-propylbenzene (0.45 g, 1.2 mmol), 4-hydroxyphenylboronic acid (0.22 g, 1.6 mmol), 1M aq. solution of sodium carbonate (5 mL, 5.0 mmol), and isobutanol (5 mL). After stirring 12 h at reflux, the reaction was cooled to RT, diluted with 30 mL of water, and extracted with ethyl acetate 2 × 25 mL. The organic phase was washed with Na_2_CO_3_ (1 M aq. Solution), dried over Na_2_SO_4_, and concentrated under reduced pressure. The residue was purified by column chromatography (DCM, then DCM/MeOH = 97/3) to give this model compound as a yield of white crystals (0.15 g, 55%).

^1^H NMR (400 MHz, CDCl_3_) δ 7.35 (d, *J* = 8.4 Hz, 2H), 7.07–7.02 (m, 2H), 6.94 (d, *J* = 8.4 Hz, 2H), 6.89 (d, *J* = 8.0 Hz, 1H), 5.23 (s, 1H, OH), 5.11 (s, 1H, OH),2.55 (t, *J* = 7.6 Hz, 2H), 1.64 (m, 2H), 0.96 (t, *J* = 7.6 Hz, 3H), (Figure S31). ^13^C NMR (101 MHz, CDCl_3_) δ 155.2, 150.3, 135.0, 130.5, 130.1, 129.7, 128.7, 127.4, 116.1, 115.4, 37.2, 24.7, 13.8, (Figure S32). HRMS (ESI) m/z: calc. for C_15_H_16_O_2_: [M + Na]^+^ = 251.1042, found 251.1038.

### 3.3. Animal Studies

All experiments adhered to regulations of DIRECTIVE 2010/63/EU on the protection of laboratory animals. The procedures with animals were reviewed and approved by the Ethics Committee of Smorodintsev Research Institute of Influenza. Animal studies were carried out on male C57BL/6 mice (8–10 weeks, 20 ± 1 g) and Wistar male rats (6–8 weeks, 200 ± 10 g) from the Nursery of Laboratory Animals (Rappolovo, Leningrad region). The animals were housed in a climate-controlled room with a 12/12-h light cycle. The subjects had ad libitum access to food and water during housing. Neuroinflammation was modeled in animals by intraperitoneal (i.p.) single injection of LPS from *Escherichia coli saline* O111:B4 (Sigma-Aldrich) at a dose of 2 mg/kg.

### 3.4. Anti-Inflammatory Screening 

In experiments evaluating the anti-inflammatory abilities of fluoroethoxy biphenyls, mice were injected with LPS an hour after the first administration of compounds **F-I–F-IV**, celecoxib, or a placebo. In total, 7 groups of 5 individuals in each were studied, including a group of intact animals. The tested compounds and celecoxib were injected in a dose of 10 mg/kg in saline containing 5% DMSO for 3 days (0.4 mL solution per animal per day). Mice were decapitated 72 h after LPS administration under xylazine–zoletil anesthesia (50 mg/kg) and the brain was immediately removed. The blood collected after decapitation was left in tubes with a serum separator for 30 min, then centrifuged at 611 *g* (3000 rpm) and 4 °C for 10 min. Blood and brain samples were frozen and stored at −20 °C.

The brain was weighed and 2 mL of ice-cold endotoxin-free PBS containing 0.1% non-ionic detergent Triton X-100 was added to the tissue samples left under ice for 10 min until homogenized with a tissue disruptor. After centrifugation for 5 min, supernatants were collected and stored at −20°C followed by measurements of cytokines and TBARS levels by ELISA in the serum and brain.

#### 3.4.1. Cytokine Assay

Mouse cytokines (TNF-α and IL-6) were assayed using ELISA MAX^TM^ Standard Sets adapting the procedures recommended by the manufacturer BioLegend (San Diego, CA, USA). Briefly, captured antibodies were coated with PBS pH 9.5 as recommended by the manufacturer. Avidin-horseradish peroxidase conjugate with H_2_O_2_-3,3′,5,5′-tetramethylbenzidine (Sigma-Aldrich, St. Louis, Missouri USA) substrate was used. Plates were read by microplate spectrophotometer EPOCH2TC (Bio Tek Instruments Inc., Winooski, VT, USA). The sensitivity to all cytokines ranged from 0.01 to 500 pg/mL [38].

#### 3.4.2. Histology, Cresyl Violet Staining, and Neuronal Morphology

The morphometric assessment of the total linear density of neurons (N_all_) and the number of pyknomorphic neurons (N_pykn_) was performed on histological sections from the brain regions of the hippocampus (CA1, CA3, DG) and posterior parietal cortex. Pyknotic cells were counted using ImageJ software (NIH). There were seven experimental groups of 3 mice each. The brain was removed and fixed entirely in 10% buffered formalin solution, and coronal sections were serially cut through the hippocampus and cortex, cassetted, marked, and embedded in paraffin blocks. Brain sections 8 μm thick were stained with Nissl cresyl violet, clarified, and covered with a coverslip. They were then studied at 400× magnification on a Leica DM1000 light-optical microscope and micrographs were taken using the ADF Pro camera software.

#### 3.4.3. TBARS Assay

The supernatant diluted with buffer 1:10 was used for analysis. A total of 100 μL of tissue homogenate sample (S) or control buffer/standard solution (STD) was added to 100 μL of SDS solution according to TBARS assay Elisa kit (Cayman Chemical) and mixed. Aliquots of thiobarbituric acid solution (4 mL) were added to all of tubes, which were then boiled at 100 °C for one hour, and centrifuged at 1600 g for 10 min at 4 °C. Each sample (150 μL) was loaded in duplicate into a 96-well plate, and the absorbance was measured at 535 nm on an EPOCH2TC.

#### 3.4.4. Statistical Analysis

Data were analyzed for Gaussian or normal distribution using the Shapiro–Wilk test. To assess significant differences between more than two groups of normally distributed data, we performed one-way ANOVA, followed by Holm–Sidak’s post hoc analyses. Kruskal–Wallis test was applied to non-normally distributed data, followed by Dunn’s post hoc analyses. All statistical analyses were performed using GraphPad Prism, Version 8.2.1.

### 3.5. In Vitro COX-1/2 Inhibition Assay

The inhibitory potency of **F-IV** for COX-1 and COX-2 was established, as reported in [18], using the COX (human) Inhibitor Screening Assay kit (Item.No. 701230, Cayman Chemical Company, Ann Arbor, MI, USA) according to the manufacturer’s instructions.

### 3.6. Relative Binding Free Energies of Ligands **F-I–F-IV** with CB-2 and COX-2

To compare binding affinities of our ligands to CB-2 and COX-2, using structures 6PT0 and 5KIR from Protein Data Bank (PDB), respectively, we employed the calculations of relative binding free energies of structurally similar ligands by the alchemical free energy perturbation (FEP) method [39,40]. The FEP approach is extensively applied in drug discovery projects today and it is more accurate than the MM/GBSA (molecular mechanics combined with generalized Born surface solvation) method we used previously [18]. All the molecular modeling and simulations were performed using Schrodinger 2020-2 software. We took prepared and equilibrated receptor and ligand structures for the FEP+ calculations [41,42].

The absolute free energies for MH and **F-IV** were calculated from experimental data available from this study and the literature [26]. In all cases, both the FEP and cycle closure correction errors did not exceed 0.23 and 0.62 kcal/mol, respectively, for all the complexes, supporting the reliability of calculations performed. A more detailed description of the calculations can be found in the Supplementary Materials.

### 3.7. Radiochemistry

#### 3.7.1. Radiochemistry Materials and Methods

The enriched [^18^O]H_2_O (>97%) for [^18^F]fluoride production was purchased from Global Research Technologies, Inc. (St.- Petersburg, Russia). Cartridges containing tetramethylammonium anion exchange resin (QMA light, Waters) were activated with 0.5 M K_2_CO_3_ (10 mL) and water (15 mL) immediately before use. Lipophilic sterile membrane filters Millex GV (0.22 μm) were from Millipore. “QMA eluent” was prepared as described in [43]: kryptofix 2.2.2 (99 ± 1 mg, 0.25 mmol) and K_2_CO_3_ (20 ± 1 mg, 0.12 mmol) were dissolved in Milli Q deionized water (0.90 ± 0.05 mL) and acetonitrile (20 mL).

**[^18^F]F-IV** and [^11^C]MPbP were isolated from crude reaction mixtures by semi-preparative HPLC system available on Tracerlab FX C Pro module (GE, Chicago, Illinois, USA), consisting of a SYCAM S1122 pump, 2 mL injection loop, UV detector KNF, and β-radioactivity flow detector. Column XBridge Prep C18 5 µm 10×250 mm was used with EtOH/H_2_O (70:30 *v*/*v*) as the mobile phase at a flow rate of 2.0 mL/min. The radioactivity was measured on a Curiementor 2 isotope calibrator (PTW Freiburg, Germany), and a gamma counter (BDBSZ-1eM, Moscow, Russia) was used in biological studies. Radioactivity data were decay-corrected.

Analytical radio-HPLC was performed on a Gilson system consisting of a Gilson 305 pump, Rheodyne-7125 injector with a 20 μL loop, and Gilson-116 UV detector (set to 280 nm) connected in series with a Beckman-170 radiodetector at a delay of 0.5 min. Conditions: column: XBridge C18 5 µm, 150×4.6 mm (Waters, Milford, Massachusetts, USA); eluent: MeCN/H_2_O (60:40 *v*/*v*); flow rate: 1.3 mL/min.

#### 3.7.2. Radiosynthesis of [^18^F]FEtPbP and [^11^C]MPbP 

Radioligand [^11^C]MPbP was prepared as previously reported [18]. Briefly, [^11^C]carbon dioxide was obtained by a nuclear reaction ^14^N(p, alpha)^11^C by irradiation of a gas target in PETtrace cyclotron (GE Healthcare) with a proton beam (16.5 MeV, 50 μA) in 20 min. It was transferred by a nitrogen flow to the reactor of a home-made fully automated module operated by the VARIOCONTROL program (Scintomics, Germany). [^11^C]CH_3_I was obtained by so-called “wet” method, namely the reaction of [^11^C]carbon dioxide with lithium aluminum hydride followed by hydrolysis by 57% hydroiodic acid. The resulting [^11^C]CH_3_I was bubbling in the vessel with **15** (~2 mg, 6 μmol) in acetone (1.2 mL) with 15 µL of 0.7 M NaOH in EtOH/H2O (50/50, *v*/*v*). [^11^C]methylation of **15** was carried out for 5 min at RT, and the resulting intermediate **16** was hydrolyzed with 0.3 mL HCl conc. (12 N) for 1 min at 70 °C, followed by dilution with water and purification by HPLC. Total synthesis time was 30 min. Radiochemical yield of [^11^C]MPbP was 22 ± 5% (*n* = 5) based on [^11^C]CH_3_I radioactivity.

**[^18^F]F-IV** was obtained by [^18^F]fluoroethylation of **15** using [^18^F]FEB synthon followed by hydrolysis of the intermediate **17** and HPLC purification of the crude reaction mixture.

Radionuclide [^18^F] was produced via ^18^O(p,n)^18^F nuclear reaction by proton irradiation (16.5 MeV, 30 µA) of the enriched [^18^O]H_2_O target in PETtrace cyclotron. N.c.a [^18^F]fluoride absorbed on QMA light Sep-Pak cartridge was eluted with 2 mL of “QMA eluent” to reactor of home-made module for [^18^F]FEB preparation as described before [27]. Briefly, 2-bromoethyl tosylate (~7.5 mg, 30 μM) in o-DCB (1 mL) was added to the eluent evaporated to dryness at 110 °C to prepare [^18^F]FEB. The obtained [^18^F]FEB was transferred with nitrogen flow (15–20 mL/min) into a vessel for [^18^F]fluoroethylation and bubbled for 15 min at RT. The precursor **15** solution was prepared as follows: **15** (~1.5 mg, 4 μmol) was dissolved in DMSO (0.7 mL) and 12 µL of 0.7 M NaOH in EtOH/H_2_O (50/50, *v*/*v*) or 350 µL of 0.025 M potassium methoxide in DMSO, respectively. The precursor/base molar ratio was kept as 1/2. Then, [^18^F]fluoroethylation reaction was performed at 110 °C for 10 min. A model reaction of [^18^F]FEB with *5-propyl-1,1′-biphenyl-2,4′-diol* was carried out under the same conditions with NaOH as base. The intermediate **17** was hydrolyzed with 0.3 mL of HCl conc. during 10 min at 110 °C. After cooling, the crude reaction mixture was diluted with 0.5 mL of water, **[^18^F]F-IV** was purified by semi-preparative HPLC, and the product fraction was collected at 12–16 min in a dilution flask containing 18 mL of sterile phosphate buffer pH = 7.4. The formulated solution of **[^18^F]F-IV** in 20 mL PBS containing 7% EtOH (*v*/*v*) was passed through a sterile membrane filter into a sterile bottle to provide 2050 ± 540 MBq (50 mCi ± 15 mCi); radiochemical yield was 35 ± 9% (*n* = 12) based on [^18^F]FEB radioactivity. Total synthesis time was 60 min.

Radiochemical and chemical purity was determined by analytical reversed-phase HPLC under previously described conditions (Section 3.7.1). The R_t_ value of **[^18^F]F-IV** was 6.5–6.8 min (Figure 5D). The identity of **[^18^F]F-IV** was confirmed by co-injection with non-radioactive **F-IV** reference.

#### 3.7.3. Plasma-Stability Study and logD_7.4_ Determination for **[^18^F]F-IV**

The stability of **[^18^F]F-IV** was investigated in human plasma at 37 °C in the range from 0 to 60 min according to a modified protocol [44]. Phosphate-buffered saline (PBS, 0.15 M, pH 7.4) was used as a control. **[^18^F]F-IV** formulated solution (10 µL, ~1 MBq) was added to tubes with PBS (300 µL) and human plasma (300 µL). The mixture was vortexed and incubated at 37 °C. An aliquot of 100 µL from the sample was taken at different time points (0, 10, 20, 40, and 60 min) and mixed with 100 µL ice-cooled acetonitrile and centrifuged (3 min, 4500× *g*). The supernatants were analyzed by radio-HPLC using XBridge C18 analytical column.

The partition coefficient of **[^18^F]F-IV** (log D_7.4_) was determined by the shake-flask method [45] for measuring the distribution of radioactivity between 1-octanol and phosphate buffer pH 7.4. Into a tube containing 10 µL of **[^18^F]F-IV** (~1 MBq), 1.5 mL of pre-saturated 1-octanol and 1.5 mL of phosphate buffer were added and vortexed for 15 min. Then, the tube was centrifuged at 3000 g for 5 min to ensure complete separation of the phases. Then, aliquots of 500 μL were taken from each phase into separate tubes and the radioactivity was measured in a γ-counter. The measurements were performed in triplicate. The log D_7.4_ value was calculated as follows: log (counts per min in 1-octanol/ counts per min in PBS) for five independent experiments.

### 3.8. Ex Vivo Biodistribution

Ex vivo radioligand biodistribution was performed by direct radiometry of organs and tissue samples of Wistar rats. Each rat was injected with (12 ± 3) MBq (volume ≤ 0.2 mL) of **[^18^F]F-IV** through the tail vein under anesthetic conditions. Control rats were sacrificed at 10 (*n* = 3), 30 (*n* = 3), 60 (*n* = 3), and 120 (*n* = 3) min after injection. Twenty-four hours prior to ex vivo biodistribution studies, 6 rats received an intraperitoneal (i.p.) injection of LPS (2 mg/kg) from Escherichia coli (O111: B4) or saline (1 mL/kg). For blocking studies with celecoxib, it was i.v. administered at a dose of 10 mg/kg 30 min prior to **[^18^F]F-IV** injection in three animals.

Blood samples and organs of interest, namely, the brain (cerebral cortex, cerebellum, medulla, and pons), thymus, heart, lung, spleen, kidney, liver, bladder, and muscle, were removed and weighed. The radioactivity in each tissue sample was measured by a γ-counter to calculate the percentage of injected dose per gram of tissue (%ID/g). Data were provided as mean ± SEM values.

### 3.9. Plasma Protein Binding (PPB) Determination

The ultrafiltration method [33] was used to estimate the protein-free fraction of **[^18^F]F-IV** and [^11^C]MPbP in human and rat plasma. Equal volumes (500 μL) of plasma or PBS (control) were mixed with the formulated solution of radioligand (5 MBq,50μL) and incubated at 37 °C for 15 min. After incubation, aliquots of mixture (200 µL) were transferred to the tubes for ultrafiltration (Amicon 3K; Merck) and centrifuged at 7000 *g* for 15 min. Equal aliquots (20 μL) of ultrafiltrate (C_free_) and plasma (C_total_) were measured with γ-counter. The obtained data were corrected for the radioligand binding to the filter membrane. Filtrations were performed in duplicate. The unbound (free) fraction of the radioligand was calculated as f_u_(%) = C_free_/C_total_ × 100%.

## 4. Conclusions

Four newly synthesized 4′-*O*-(2-fluoroethyl) honokiol-like derivatives (**F-I–F-IV**) exhibited significant anti-inflammatory activities comparable or even superior to that of the selective COX-2 inhibitor celecoxib. The selected compound **F-IV** has good inhibitory activity to COX-2 confirmed by in vitro assays and by calculations of the relative free energy (ddG) binding to the enzyme. The specific binding to COX-2 of its labeled analogue **[^18^F]F-IV** was investigated in a rat model of LPS-induced neuroinflammation. The brain uptake of [**^18^F]F-IV** in LPS-treated rats was not as high as that of the previously reported [^11^C]MPbP, but only slightly higher than in intact animals. However, blocking with celecoxib significantly decreased the uptake of **[^18^F]F-IV,** indicating that the radioligand appears to bind specifically to COX-2. Plasma protein binding, in our opinion, is one of the main factors responsible for the differences in the pharmacokinetics of **[^18^F]F-IV** and [^11^C]MPbP. Additional research is currently underway to provide more information on the pharmacokinetics and tissue distribution of **F-IV** and to study the main pathways for modifying the biphenyl structure to provide a basis for the development of new anti-inflammatory agents and potential COX-2 radioligands.

## Figures and Tables

**Table 1:** In vitro COX-1 and COX-2 inhibition data.

Compound	IC_50_ (µM)		SI (Selectivity Index) ^a^	Ref
	COX-1	COX-2		
Celecoxib	12.73 >10.00	0.05 0.05	254	[25] ^b^
			>200	[18] ^c^
MH	2.40	0.06	40	[25] ^b^
MPbP	2.53	0.14	18	[18] ^c^
F-IV	4.61	0.09	48	This study ^c^

^a^ In vitro COX-2 selectivity index SI = (COX-1 IC_50_/COX-2 IC_50_)-; ^b^ values were determined using an ovine COX-1/COX-2 assay kit; ^c^ values were determined using human COX-1/COX-2 assay kit.

**Table 2:** Measured unbound (free) fraction (f_u_, %) data for **[^18^F]F-IV** and [^11^C]MPbP.

Radioligand	f_u_ in Rat Plasma, %(*n* = 3)	f_u_ in Human Plasma, %(*n* = 6)
[^18^F]F-IV	2.01 ± 0.26	0.19 ± 0.05
[^11^C]MPbP	8.02 ± 0.37	1.88 ± 0.13

## Data Availability

The data that support the findings of this study are available from the corresponding author upon reasonable request.

## Supplementary Materials

The following are available online, Figure S1: 1H NMR spectra for compound 2, Figure S2: 13C NMR spectra for compound 2, Figure S3: 1H NMR spectra for compound 4, Figure S4: 13C NMR spectra for compound 4, Figure S5: 1H NMR spectra for compound 5, Figure S6: 13C NMR spectra for compound 5, Figure S7: 1H NMR spectra for compound 6, Figure S8: 13C NMR spectra for compound 6, Figure S9: 19F NMR spectra for compound 6, Figure S10: 1H NMR spectra for compound 7, Figure S11: 13C NMR spectra for compound 7, Figure S12: 19F NMR spectra for compound 7, Figure S13: 1H NMR spectra for compound 8, Figure S14: 13C NMR spectra for compound 8, Figure S15: 1H NMR spectra for compound 9, Figure S16: 13C NMR spectra for compound 9, Figure S17: 19F NMR spectra for compound 9, Figure S18: 1H NMR spectra for compound 10, Figure S19: 13C NMR spectra for compound 10, Figure S20: 19F NMR spectra for compound 10, Figure S21: 1H NMR spectra for compound 11, Figure S22: 13C NMR spectra for compound 11, Figure S23: 1H NMR spectra for compound 12, Figure S24: 13C NMR spectra for compound 12, Figure S25: 1H NMR spectra for compound 13, Figure S26: 13C NMR spectra for compound 13, Figure S27: 19F NMR spectra for compound 13, Figure S28: 1H NMR spectra for compound 14, Figure S29: 13C NMR spectra for compound 14, Figure S30: 19F NMR spectra for compound 14, Figure S31: 1H NMR spectra for 5-propyl-1,1’-biphenyl-2,4’-diol, Figure S32: 13C NMR spectra for 5-propyl-1,1’-biphenyl-2,4’-diol.

## Sample Availability

Samples of the compounds F-I – F-IV are available from the authors.
