# Supplementary material for: Preliminary Assessment of the Anti-inflammatory Activity of New Structural Honokiol Analogs with a 4′-O-(2-Fluoroethyl) Moiety and the Potential of Their 18F-Labeled Derivatives for Neuroinflammation Imaging
Source: Molecules. 2021 Nov 1;26(21):6630. doi: 10.3390/molecules26216630 (PMC8587714; doi:10.3390/molecules26216630)
Supplement: Supplementary file 1 [file molecules-26-06630-s001.zip › molecules-1431165-supplementary.pdf]

Supporting information to

Preliminary Assessment of the Anti-inflammatory Activity of New Structural Honokiol Analogs with a 4'-*O*-(2-fluoroethyl) Moiety and the Potential of Their ^{18}F-Labeled Derivatives for Neuroinflammation Imaging

*Daria D Vaulina[1], Kira I Stosman[2], Konstantin V Sivak[2], Andrey G. Aleksandrov[2], Nikolai B Viktorov[3], Nikolay N Kuzmich[2,4], Mariia M Kiseleva[5], Olga F Kuznetsova[1], Natalia A Gomzina[1]**

[1]N.P. Bechtereva Institute of Human Brain, 197376 St.-Petersburg, Russia;

[2]Smorodintsev Research Institute of Influenza WHO National Influenza Centre of Russia, 197376, St.-Petersburg, Russia;

[3]St.-Petersburg State Technological Institute (Technical University), 190013, St.-Petersburg, Russia;

[4]I.M. Sechenov First Moscow State Medical University, 119991, Moscow, Russia;

[5]Université Laval, Québec, QC, Canada, G1V 4G2

I.NMR spectra of new fluoroethoxy MH derivatives and synthesis intermediates

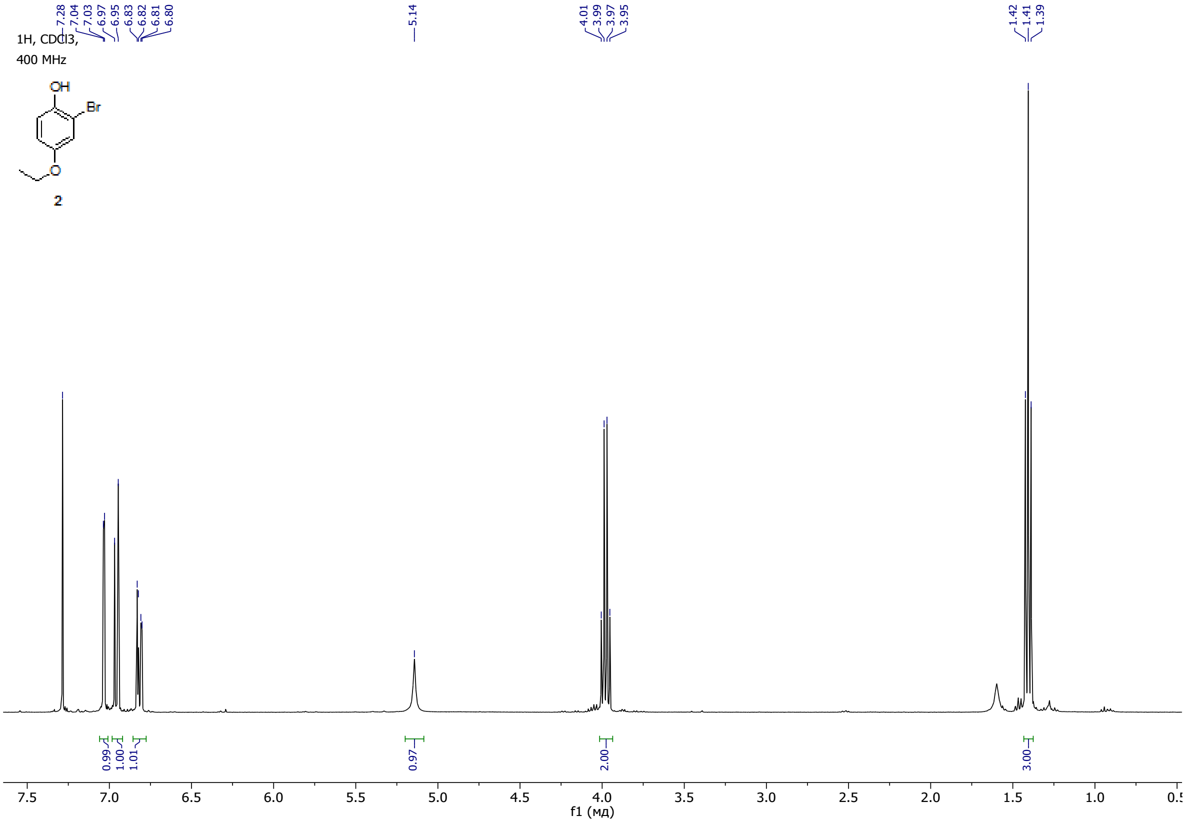

Figure S1. ^{1}H NMR spectra for compound **2**.

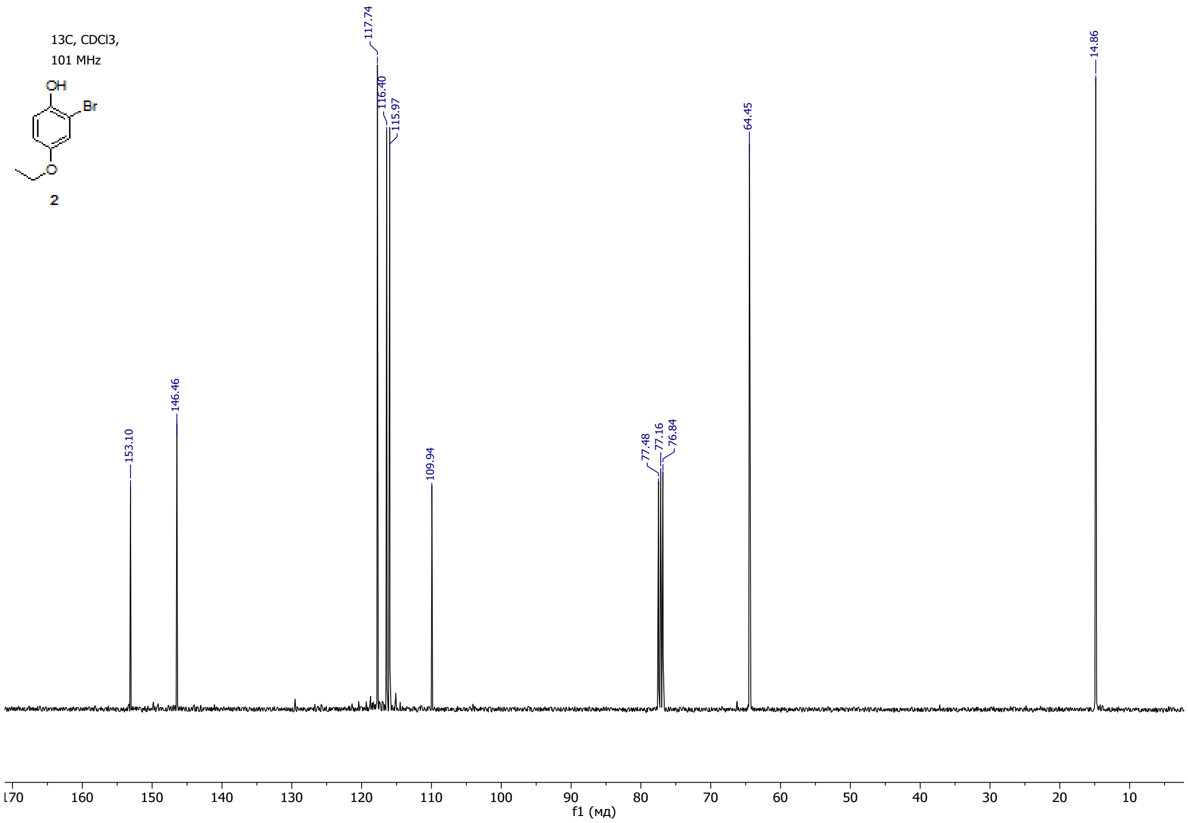

Figure S2. ^{13}C NMR spectra for compound **2**.

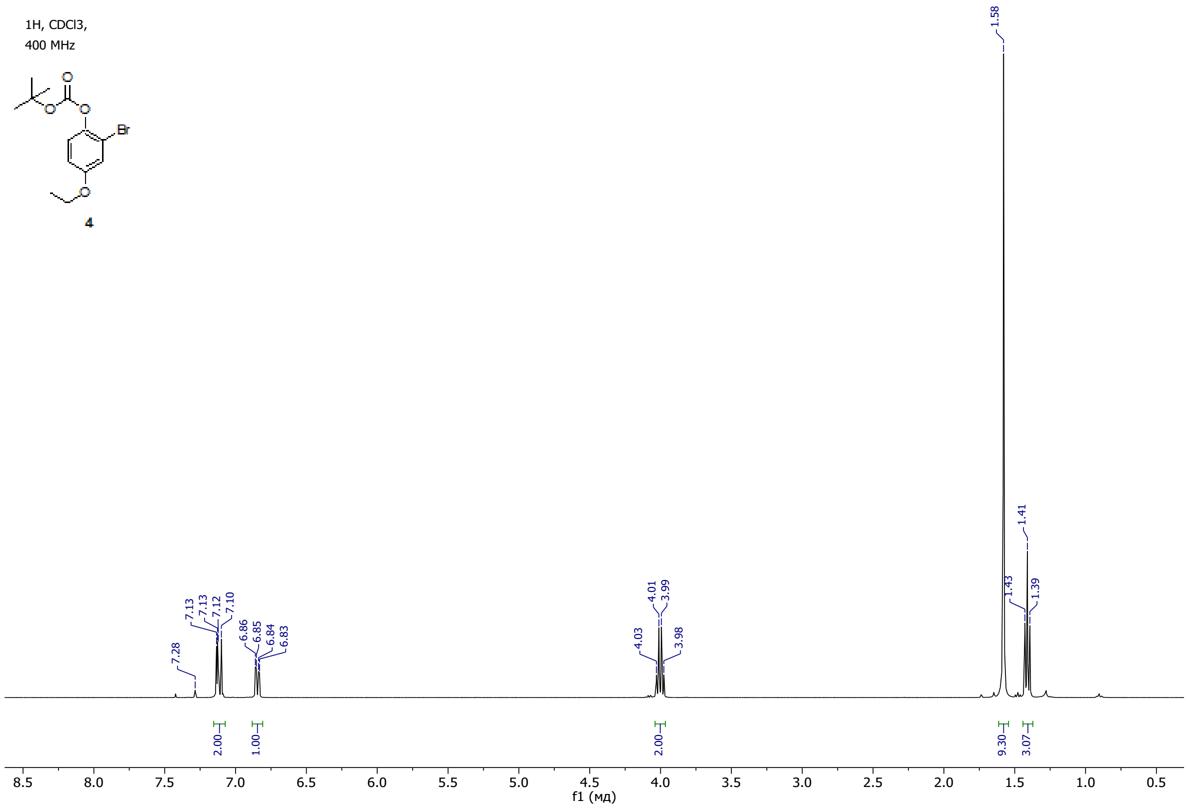

Figure S3. ^{1}H NMR spectra for compound **4**.

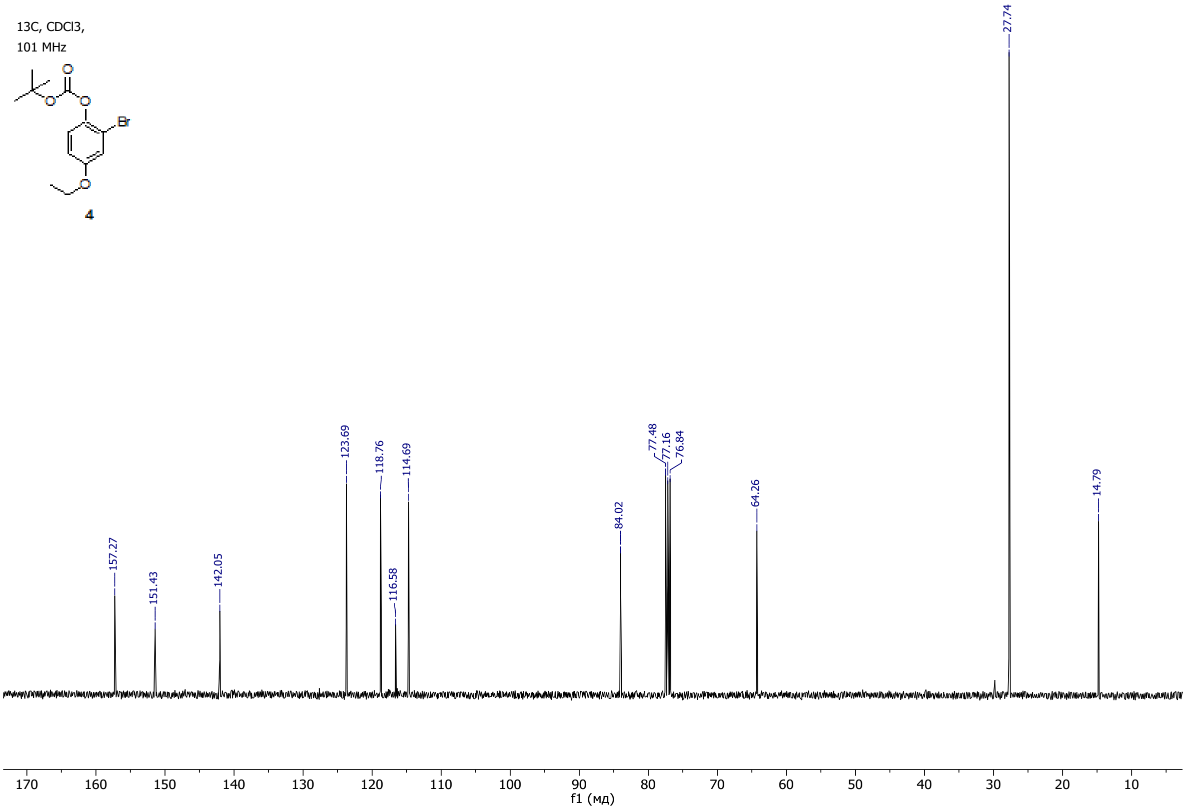

Figure S4. ^{13}C NMR spectra for compound **4**.

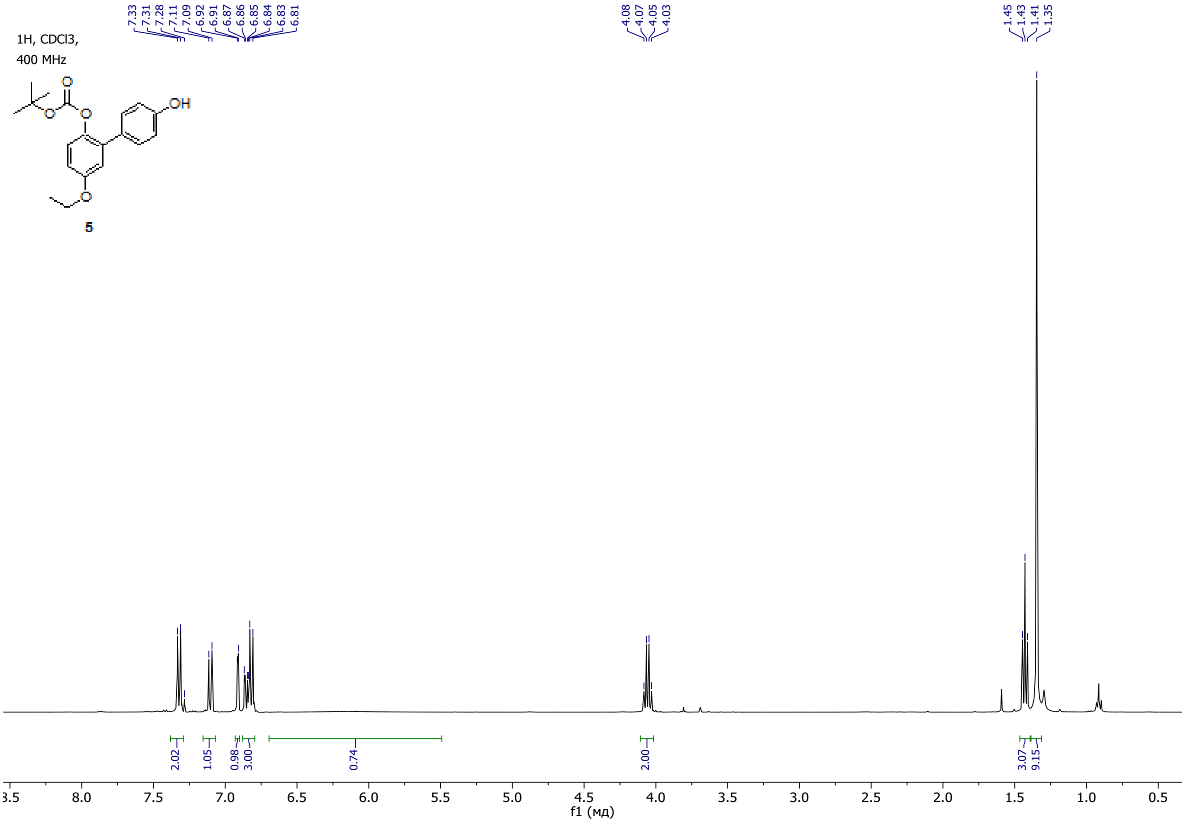

Figure S5. ^{1}H NMR spectra for compound **5**.

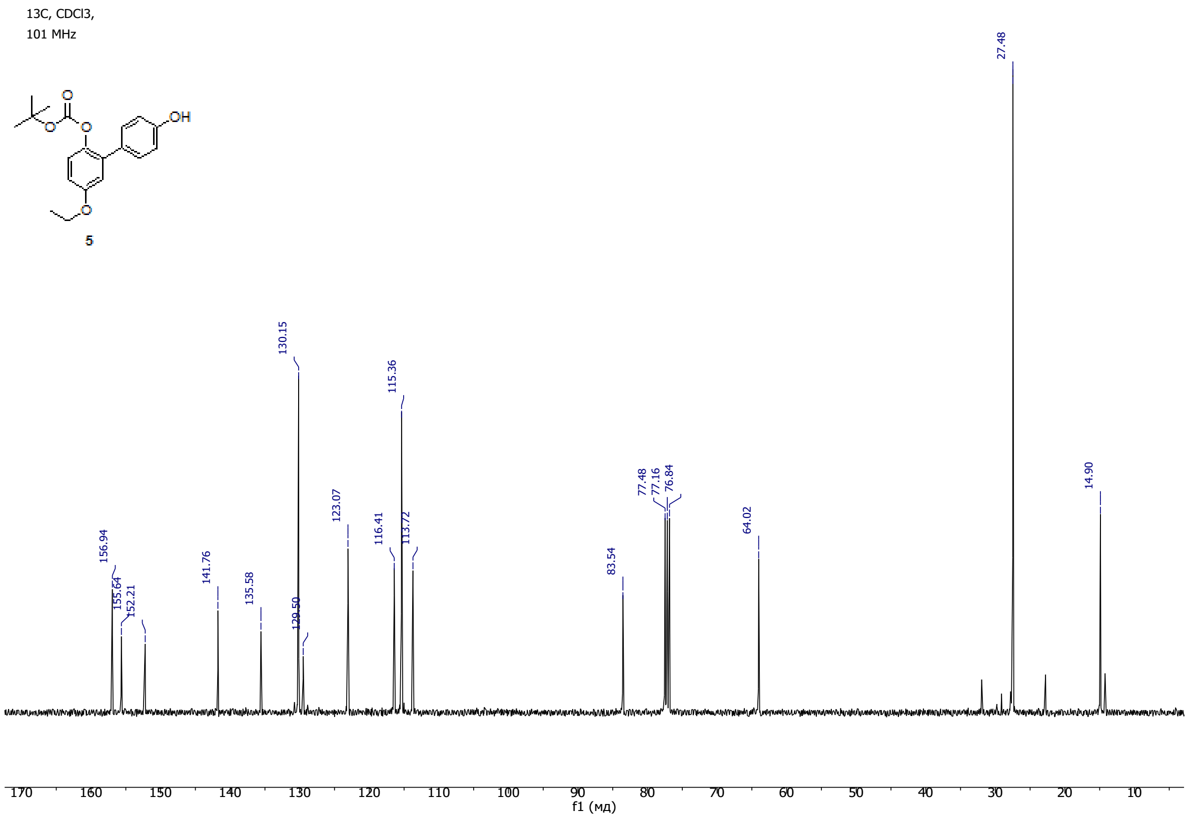

Figure S6. ^{13}C NMR spectra for compound **5**.

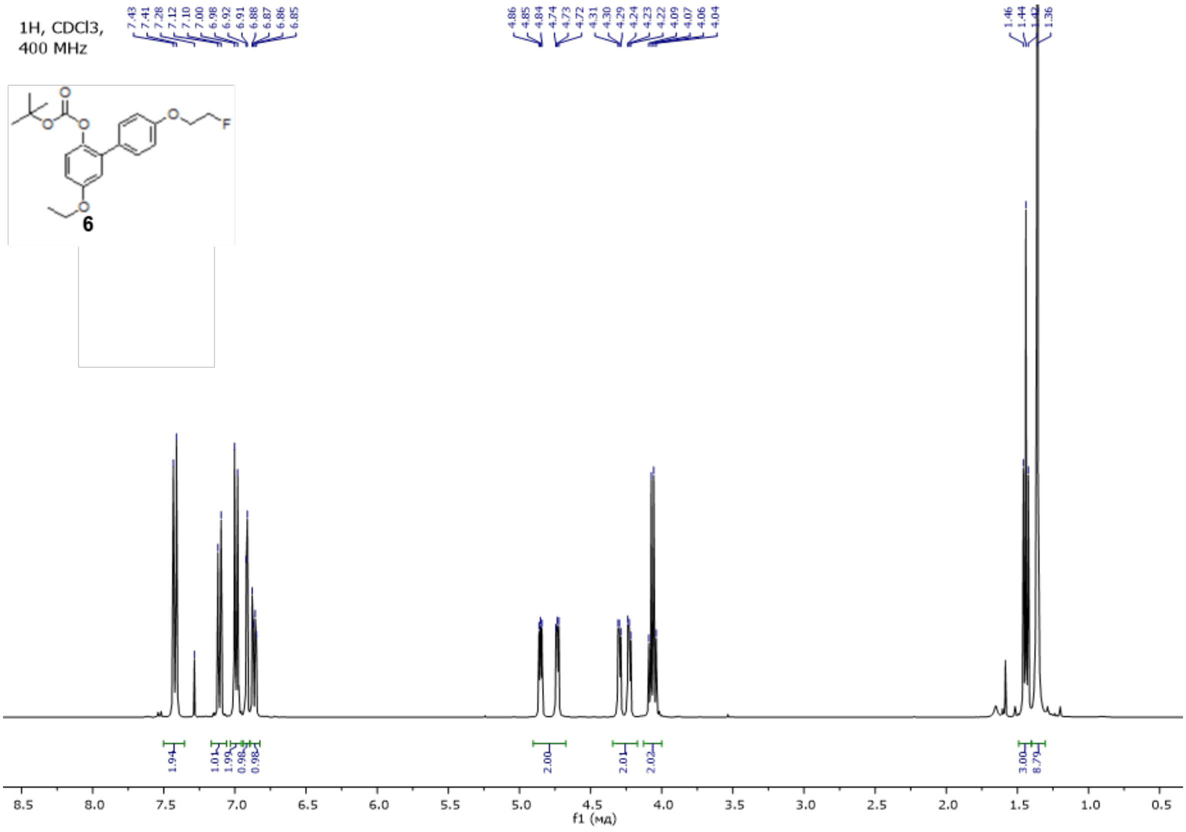

Figure S7. ^{1}H NMR spectra for compound **6**.

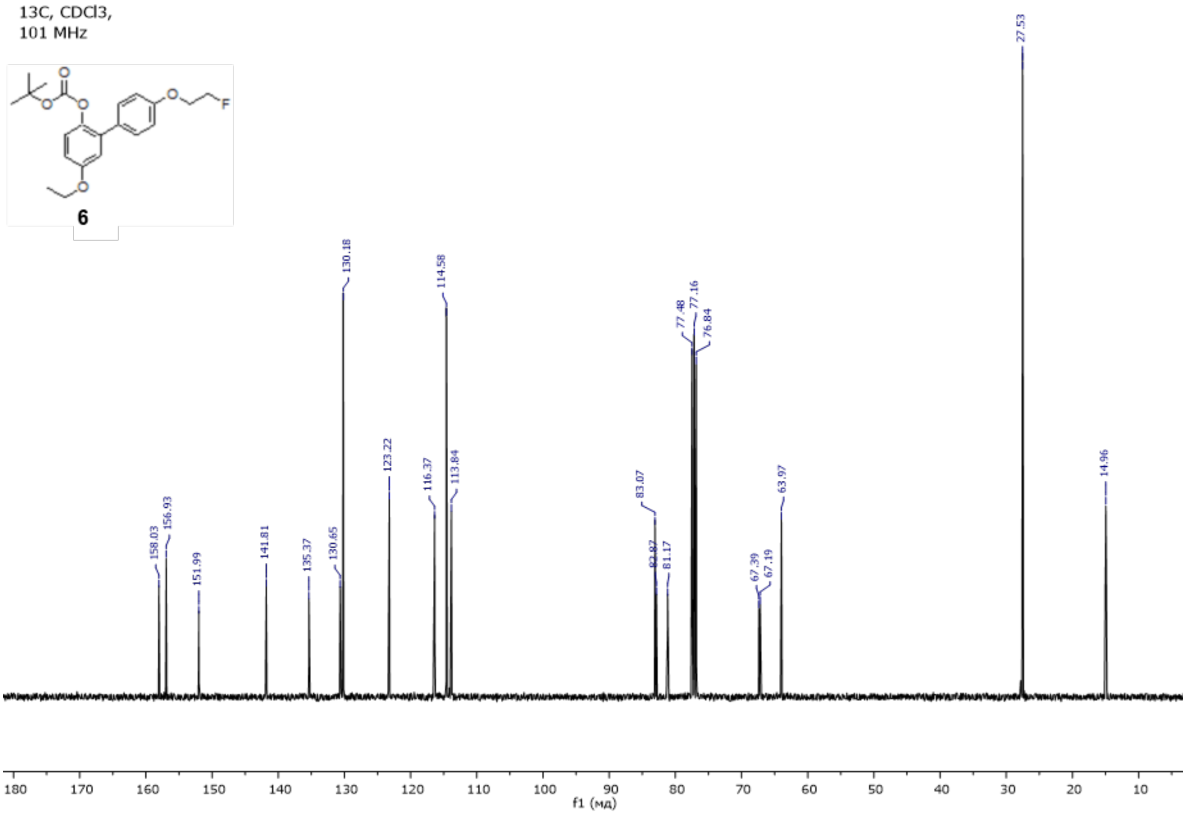

Figure S8. ^{13}C NMR spectra for compound **6**.

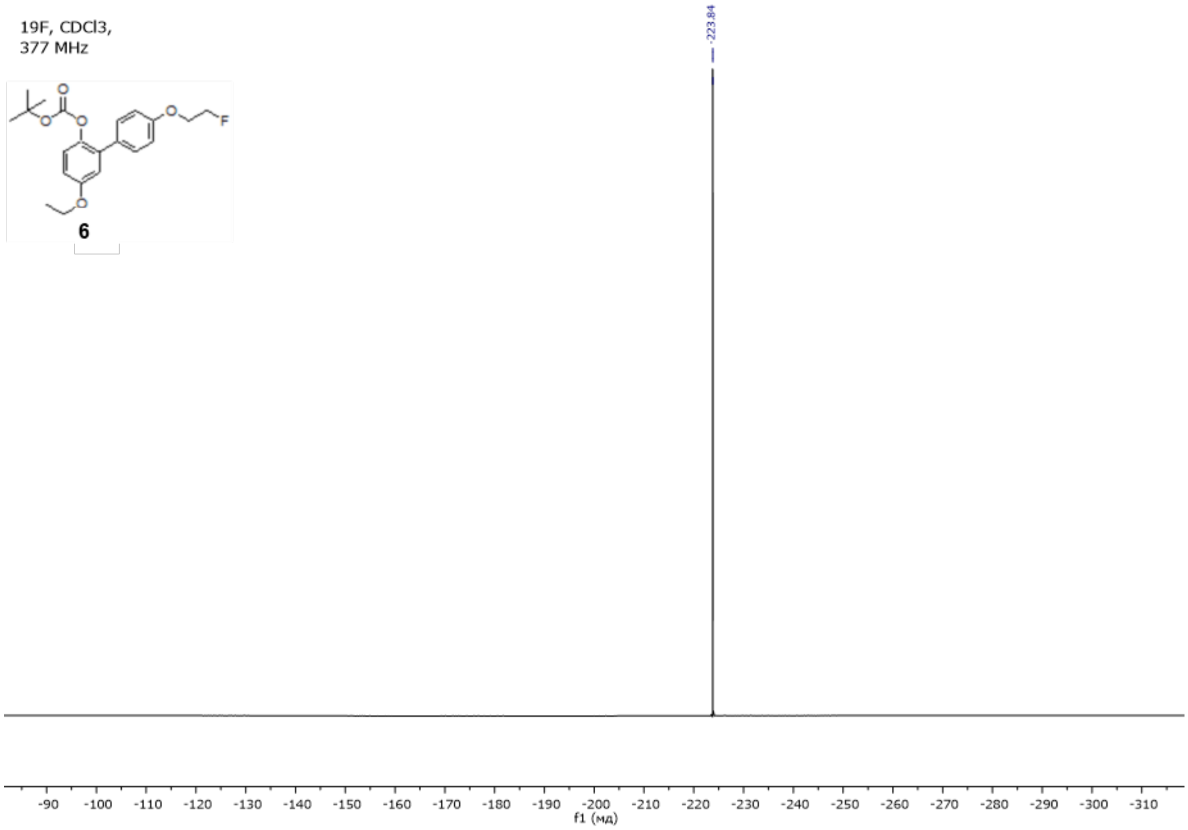

Figure S9. ^{19}F NMR spectra for compound **6**.

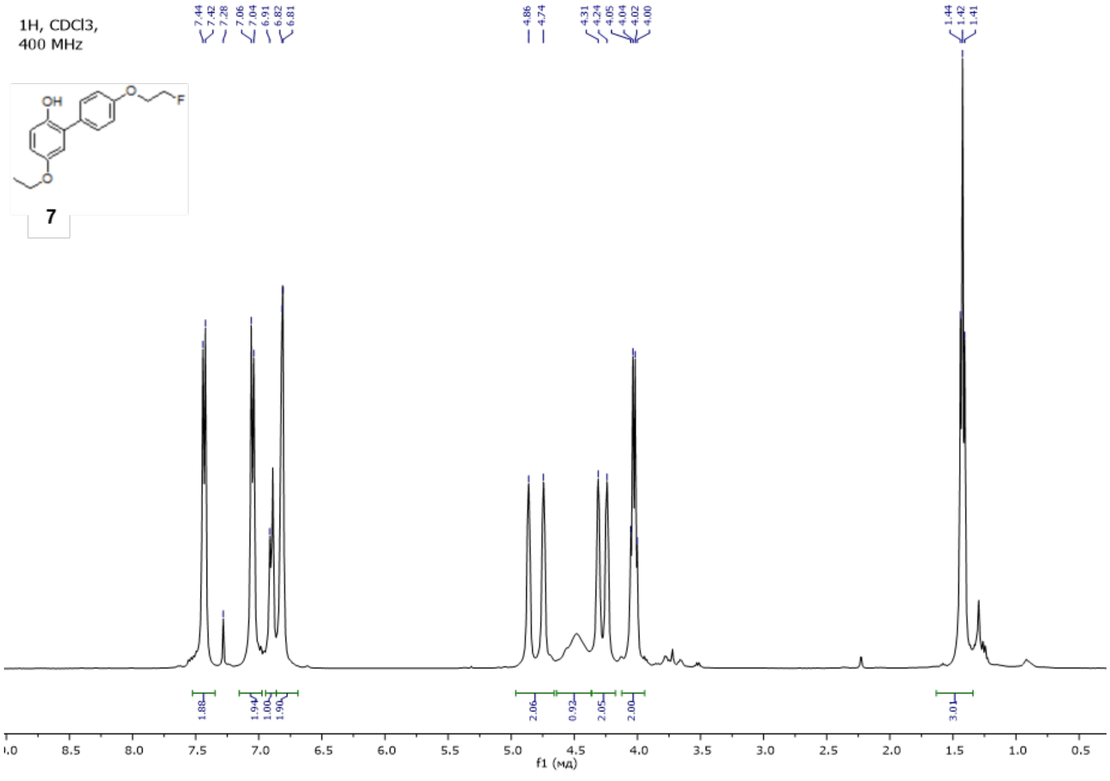

Figure S10. ^{1}H NMR spectra for compound **7**.

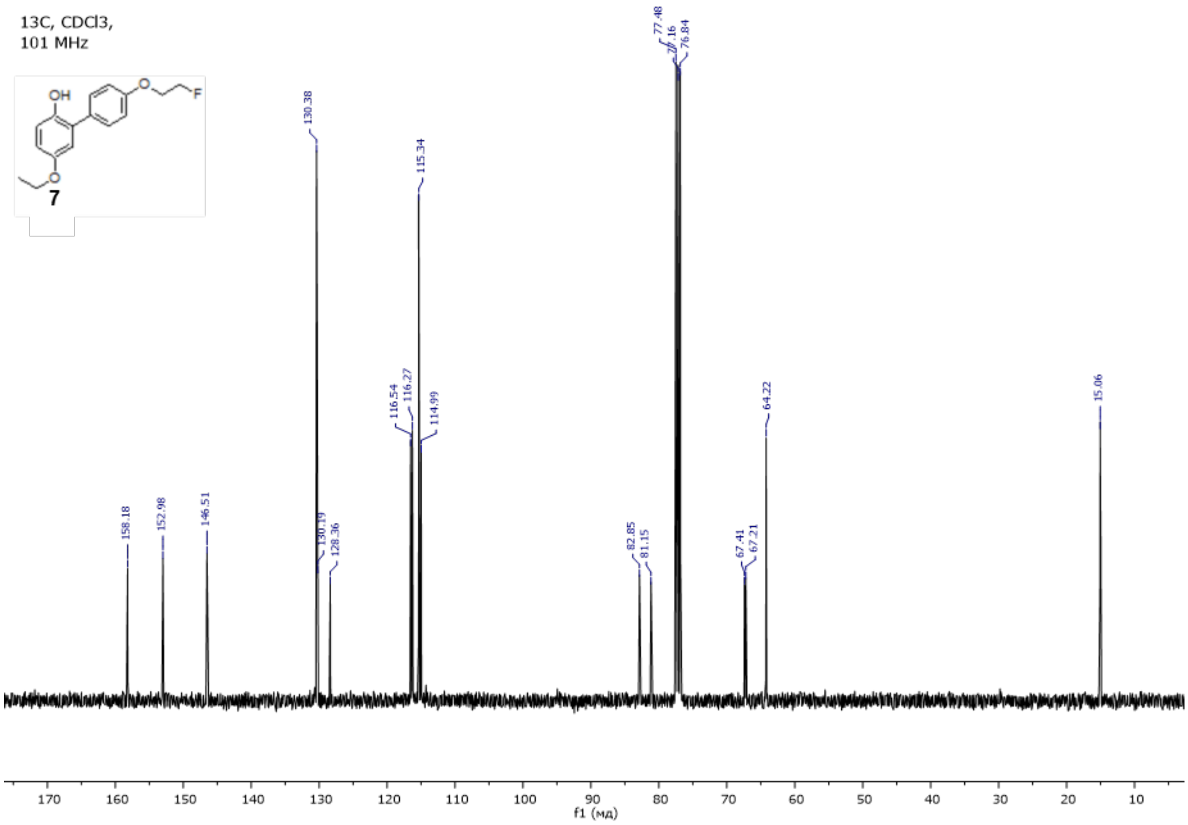

Figure S11. ^{13}C NMR spectra for compound **7**.

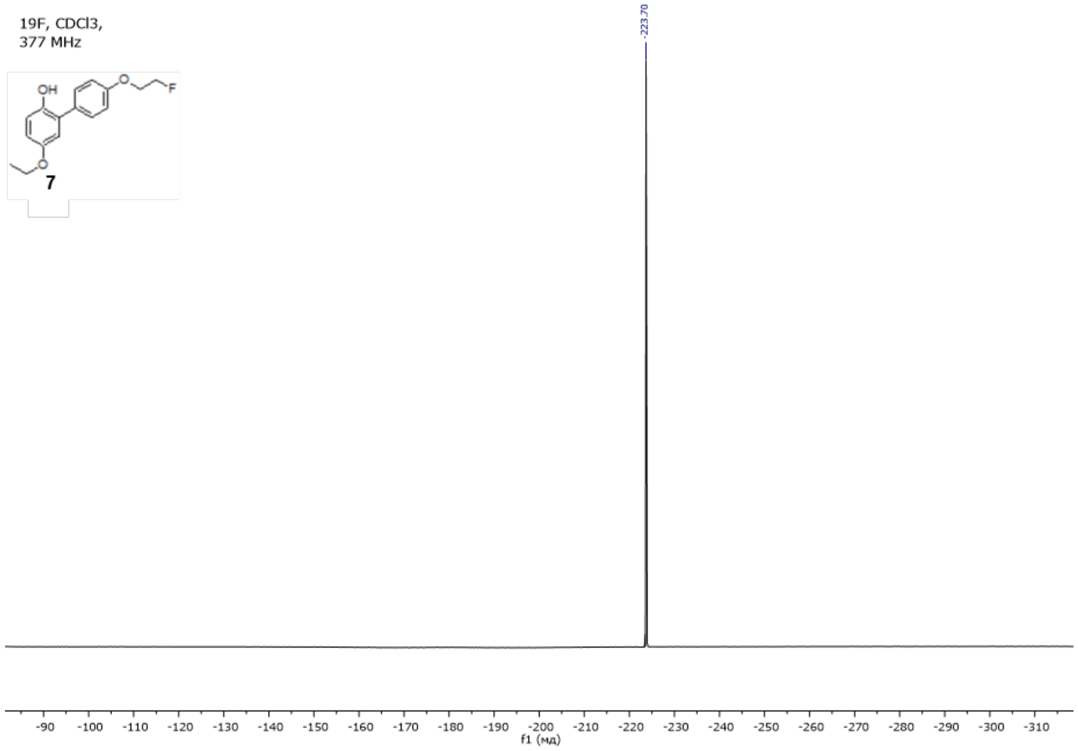

Figure S12. ^{19}F NMR spectra for compound **7**.

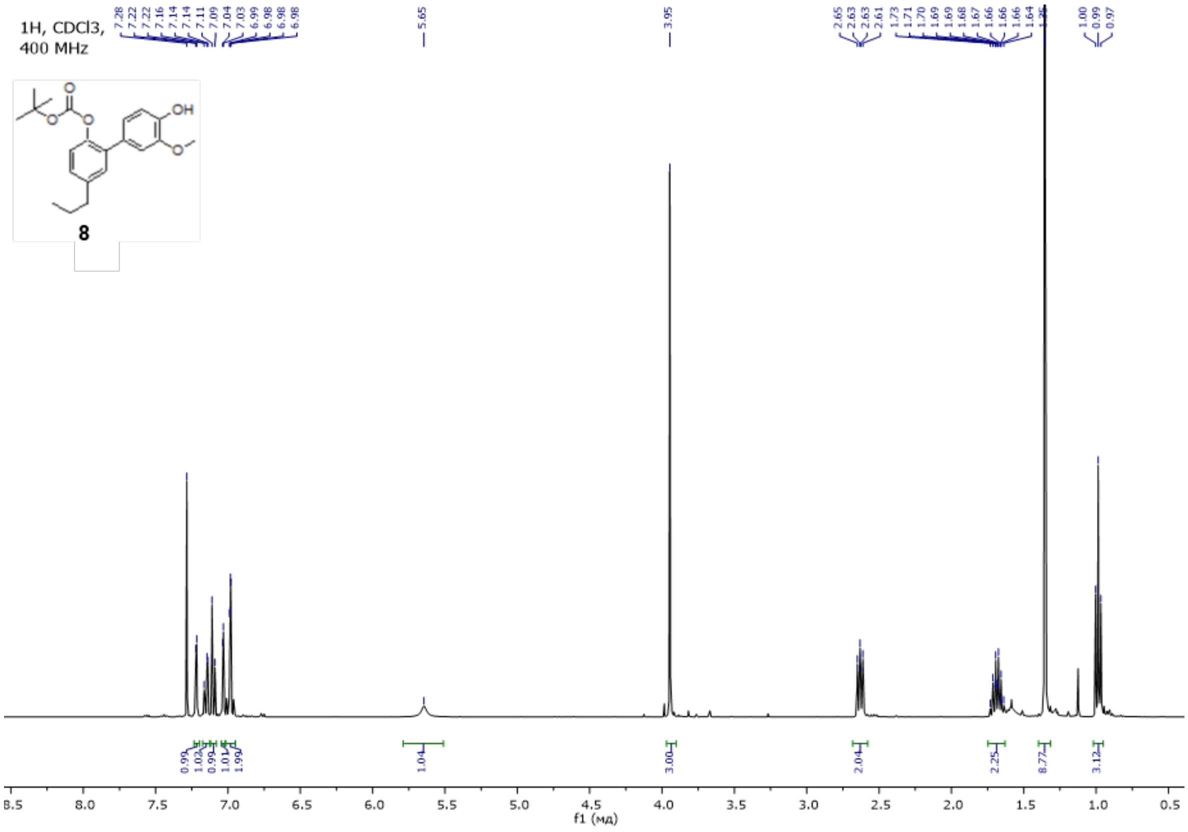

Figure S13. ^{1}H NMR spectra for compound **8**.

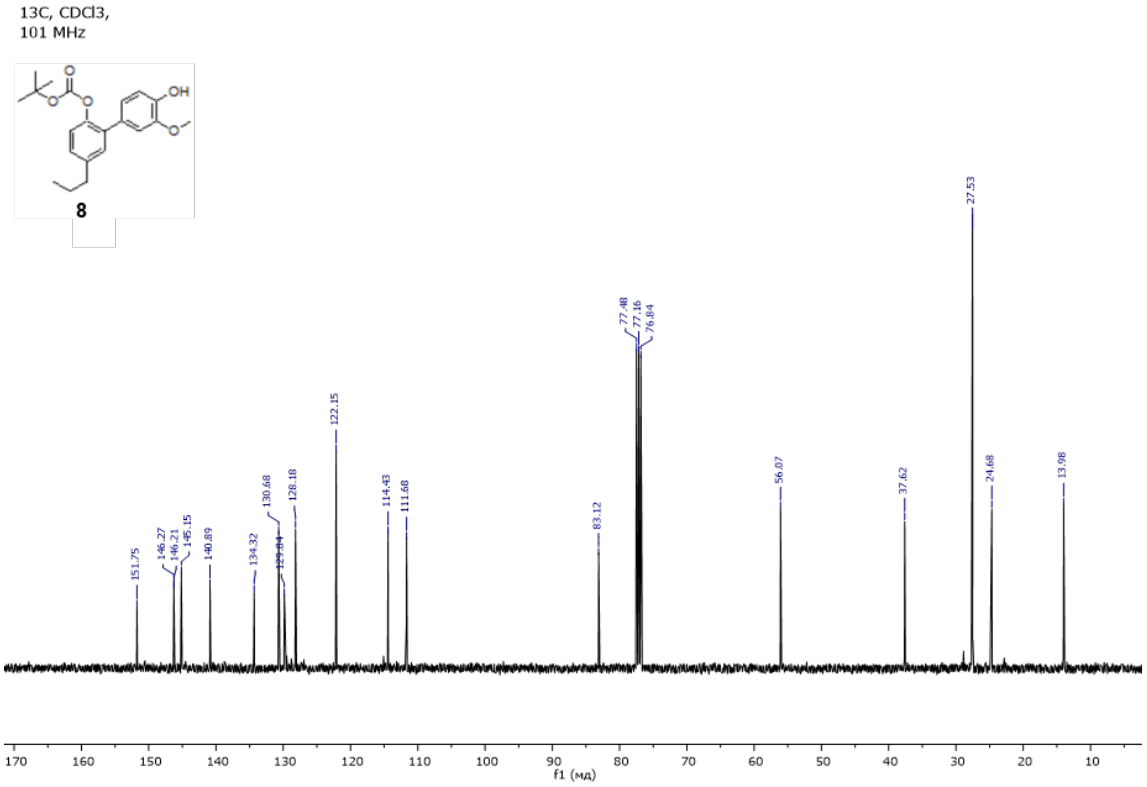

Figure S14. ^{13}C NMR spectra for compound **8**.

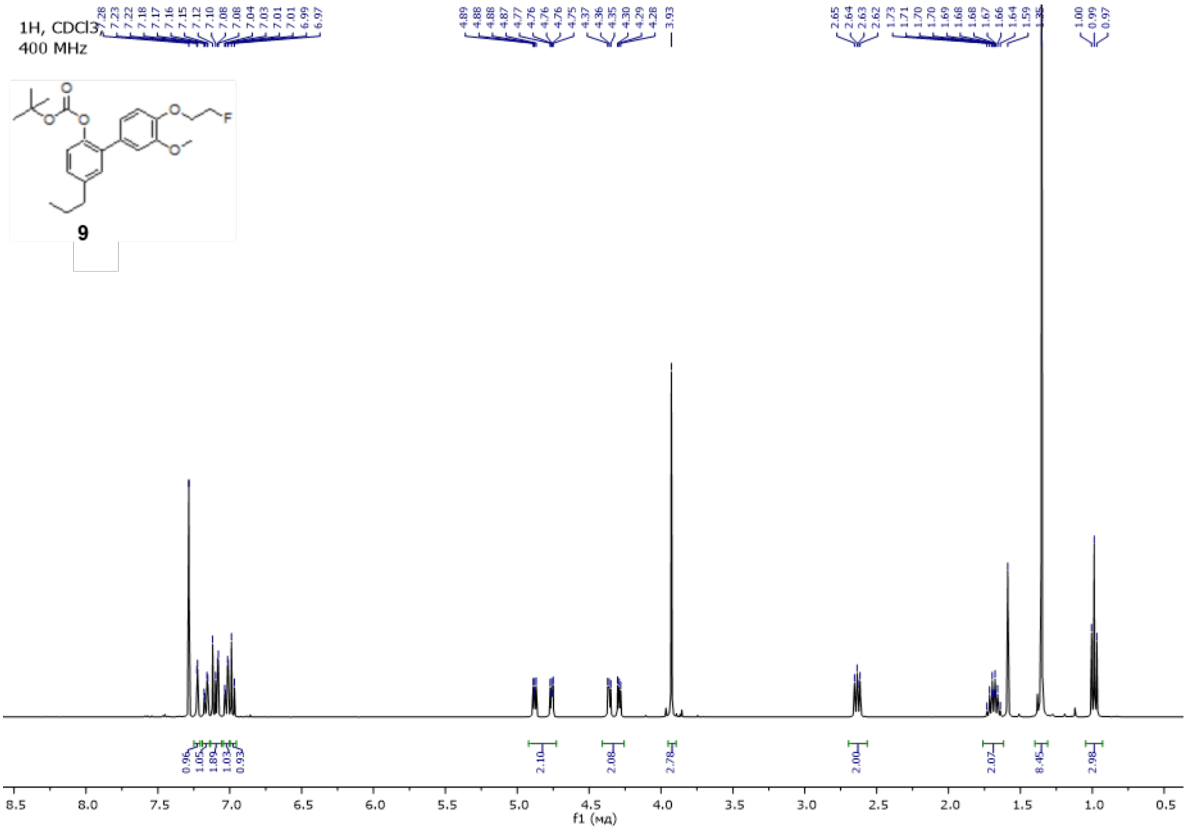

Figure S15. ^{1}H NMR spectra for compound **9**.

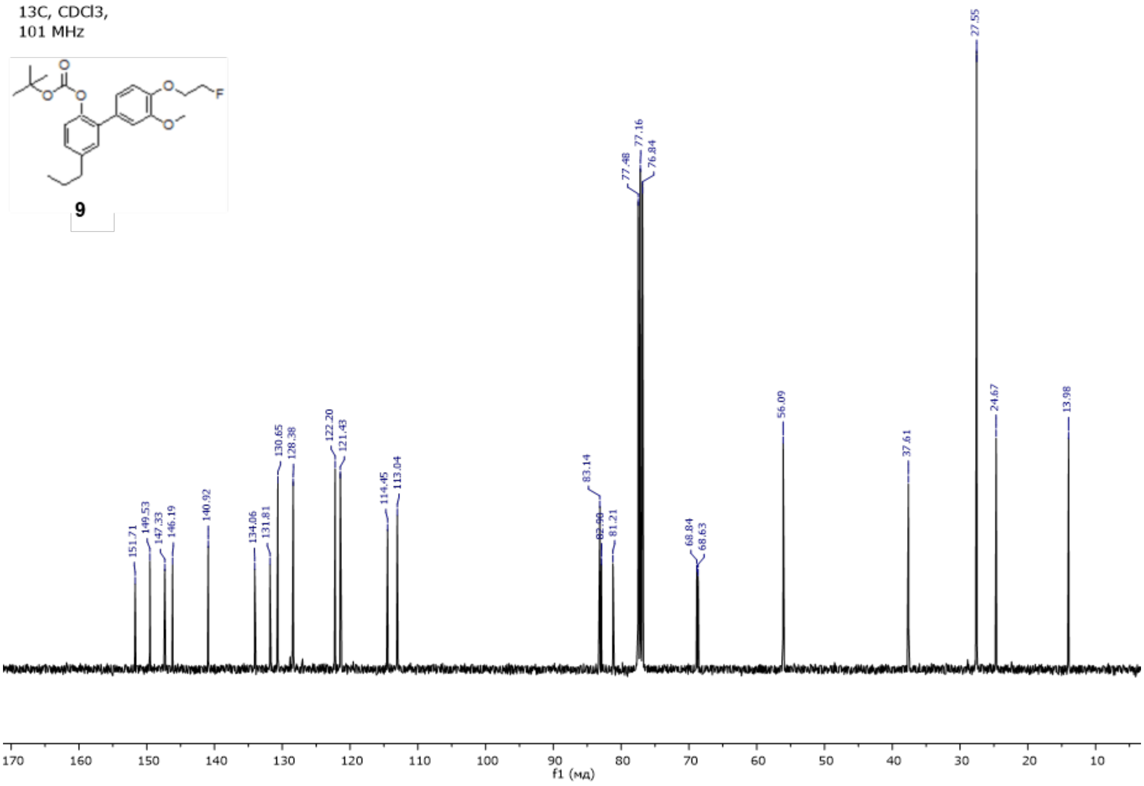

Figure S16. ^{13}C NMR spectra for compound **9**.

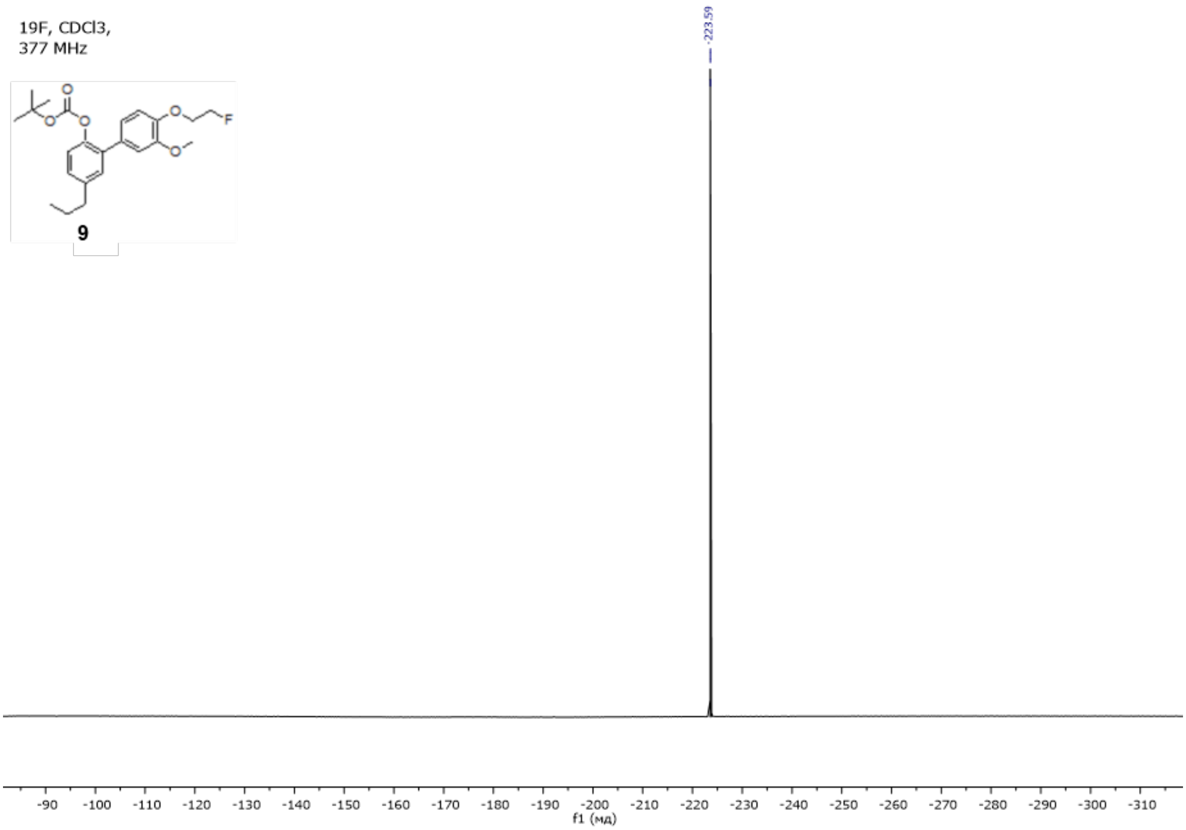

Figure S17. ^{19}F NMR spectra for compound **9**.

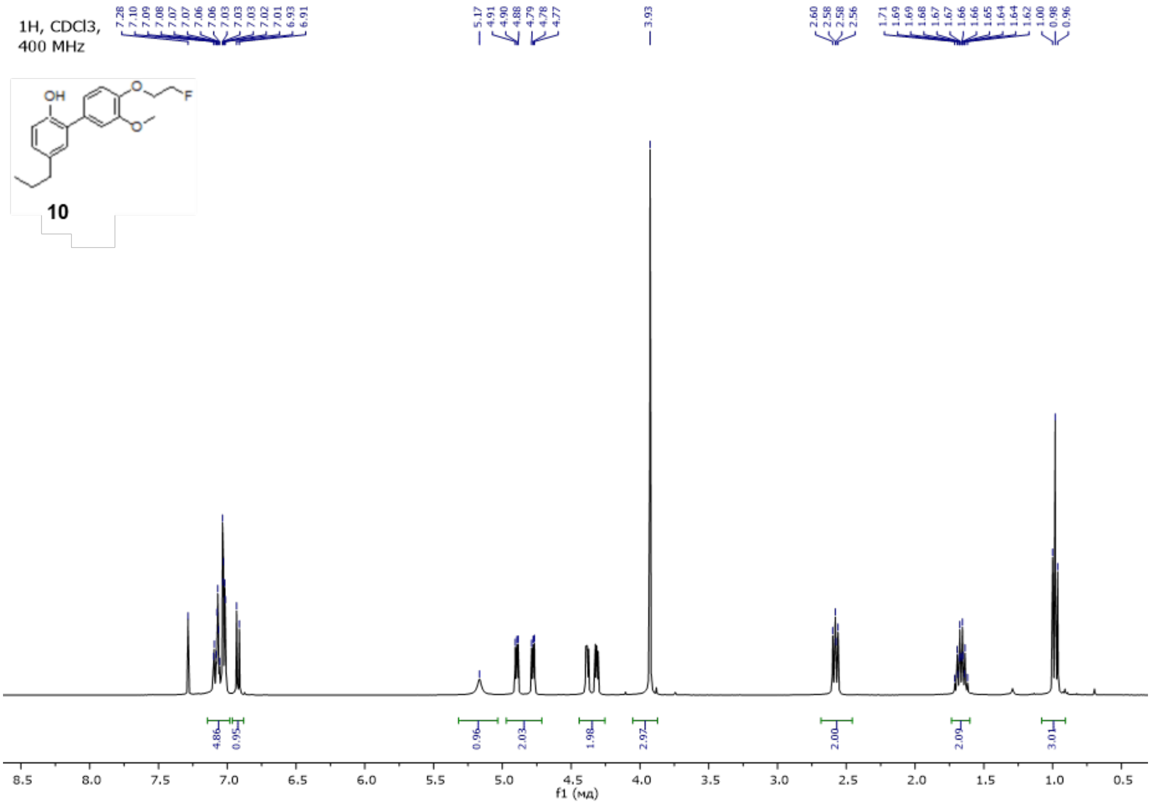

Figure S18. ^{1}H NMR spectra for compound **10**.

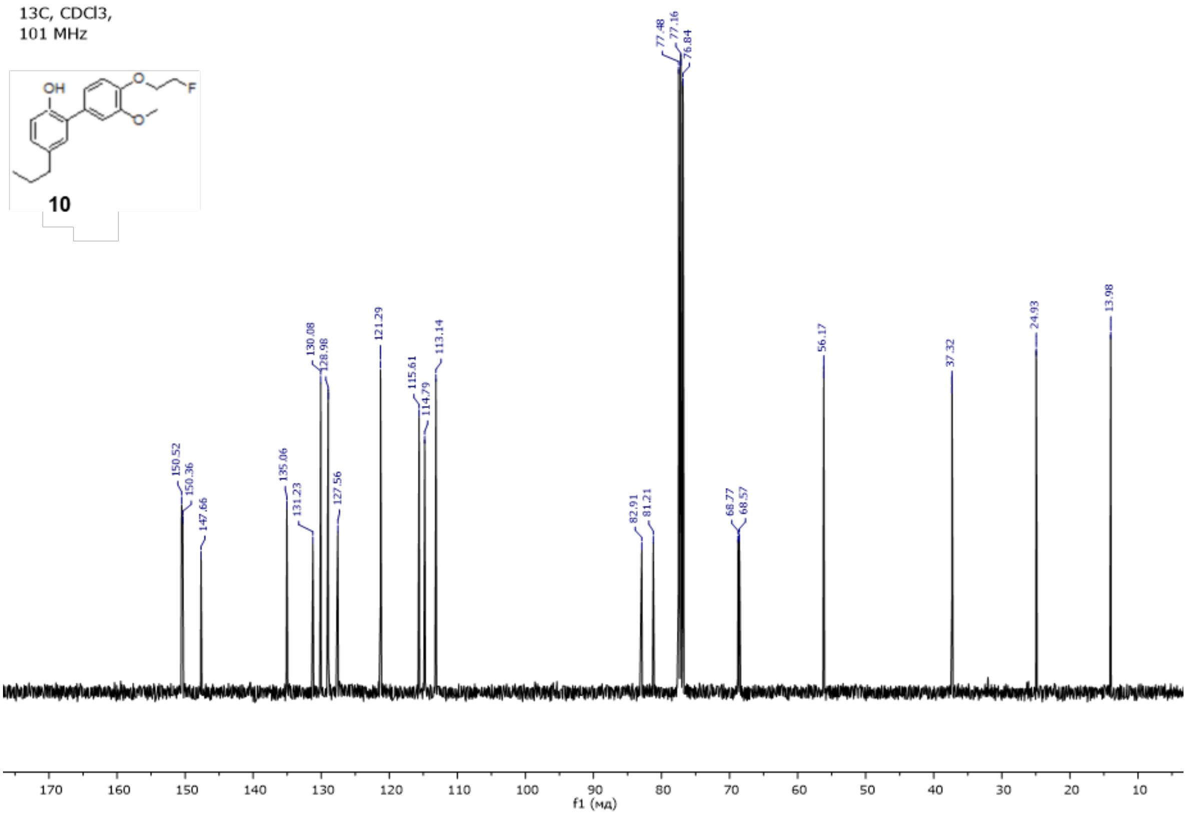

Figure S19. ^{13}C NMR spectra for compound **10**.

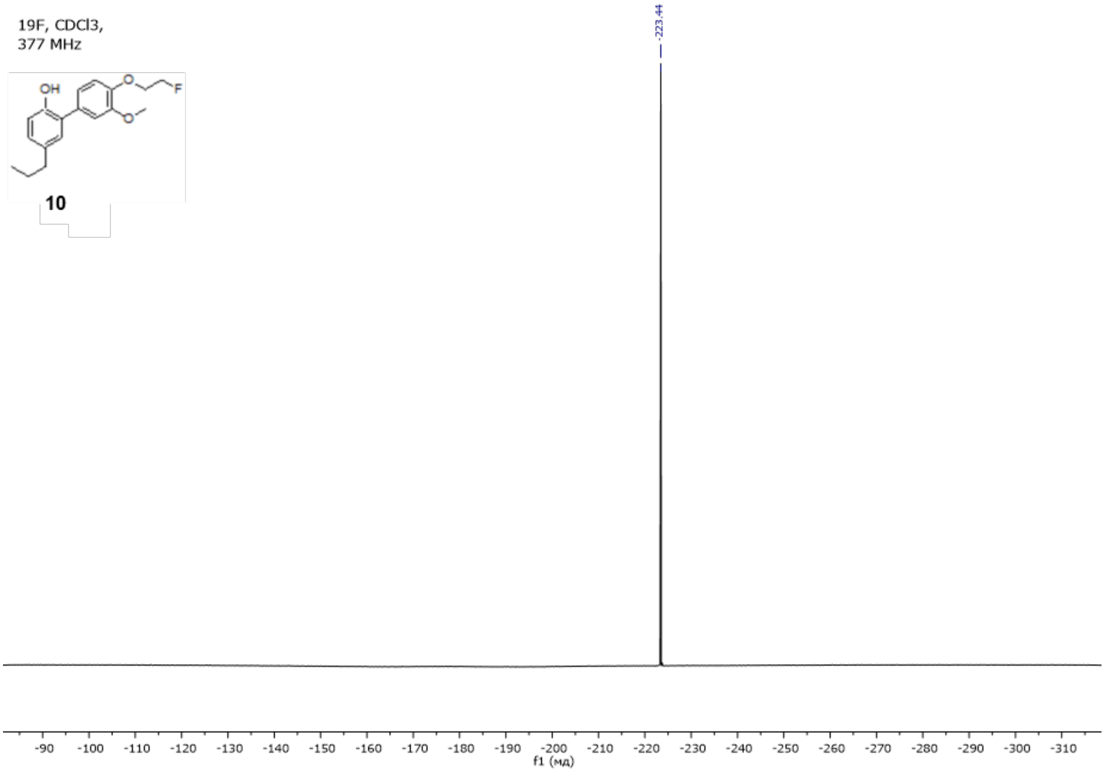

Figure S20. ^{19}F NMR spectra for compound **10**.

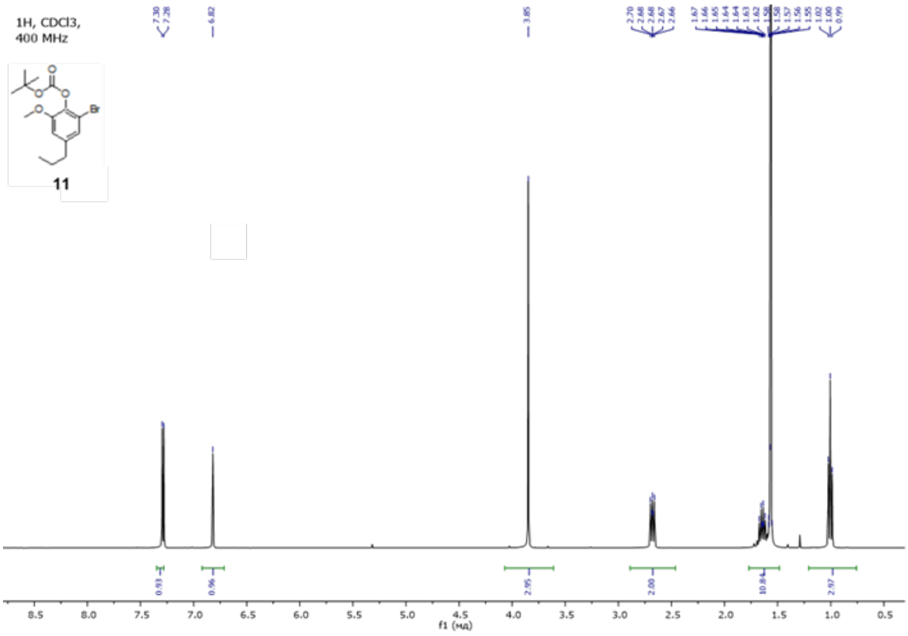

Figure S21. ^{1}H NMR spectra for compound **11**.

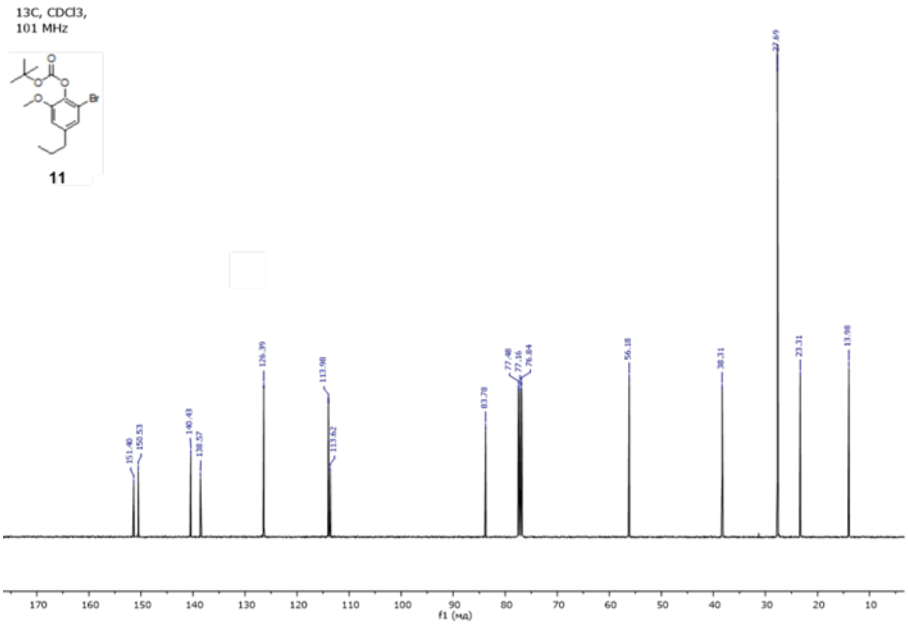

Figure S22. ^{13}C NMR spectra for compound **11**.

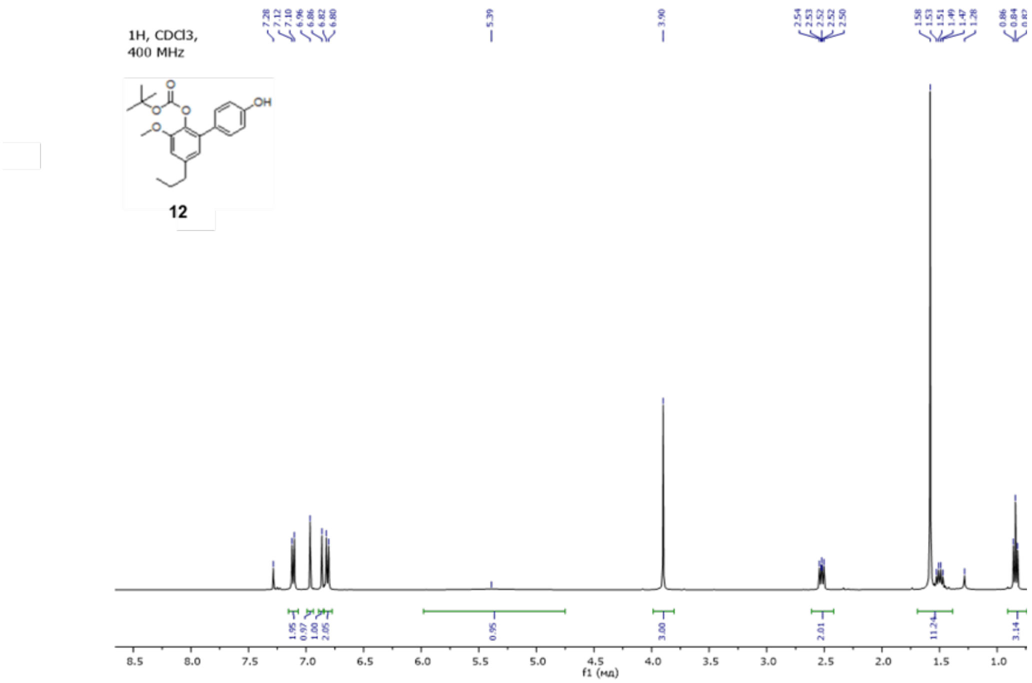

Figure S23. ^{1}H NMR spectra for compound **12**.

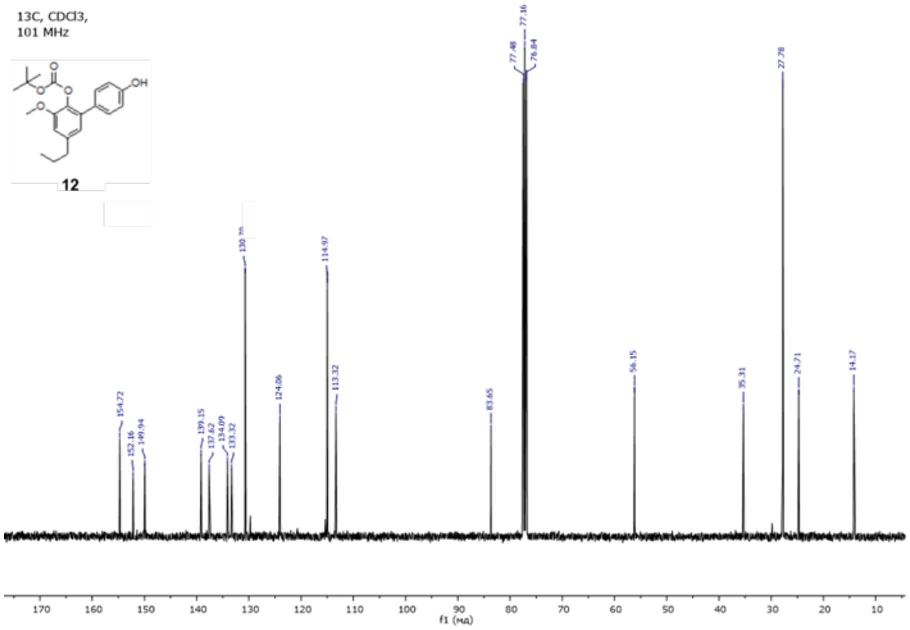

Figure S24. ^{13}C NMR spectra for compound **12**.

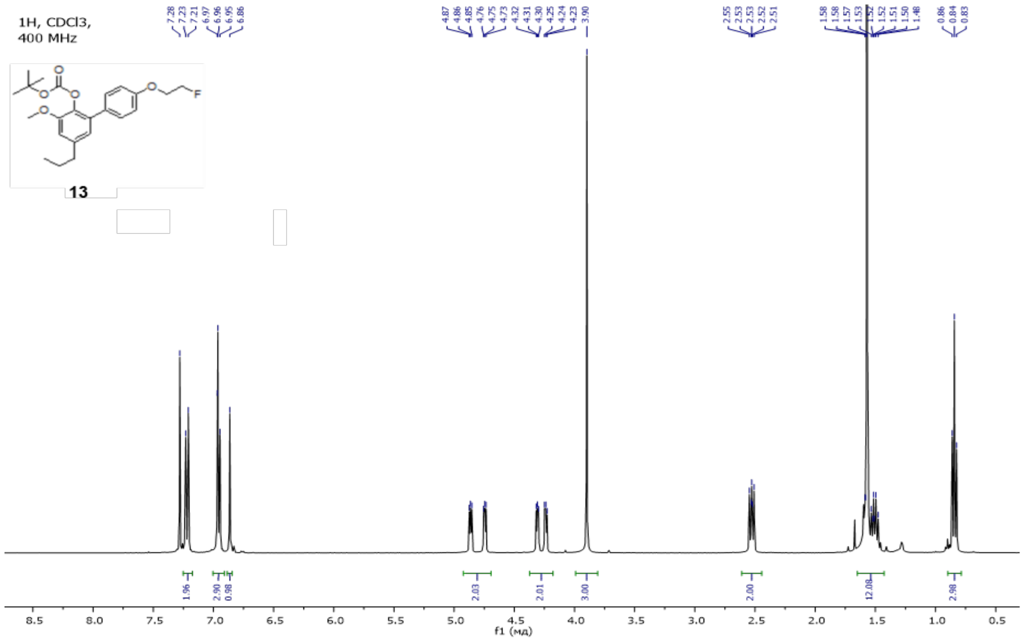

Figure S25. ^{1}H NMR spectra for compound **13**.

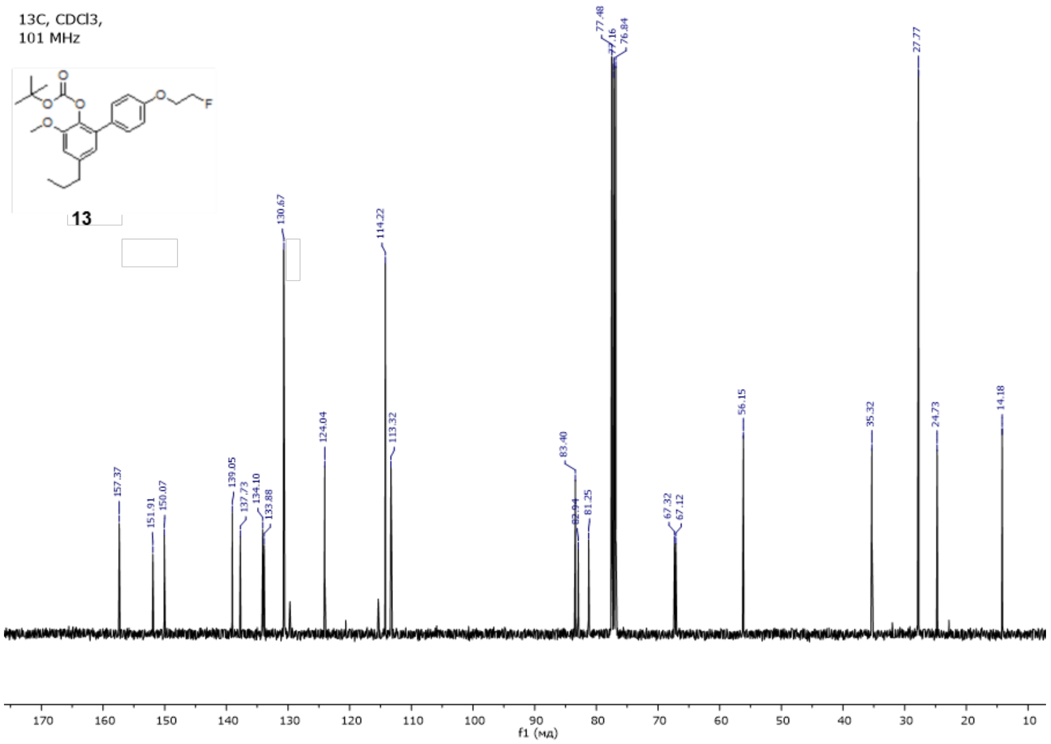

Figure S26. ^{13}C NMR spectra for compound **13**.

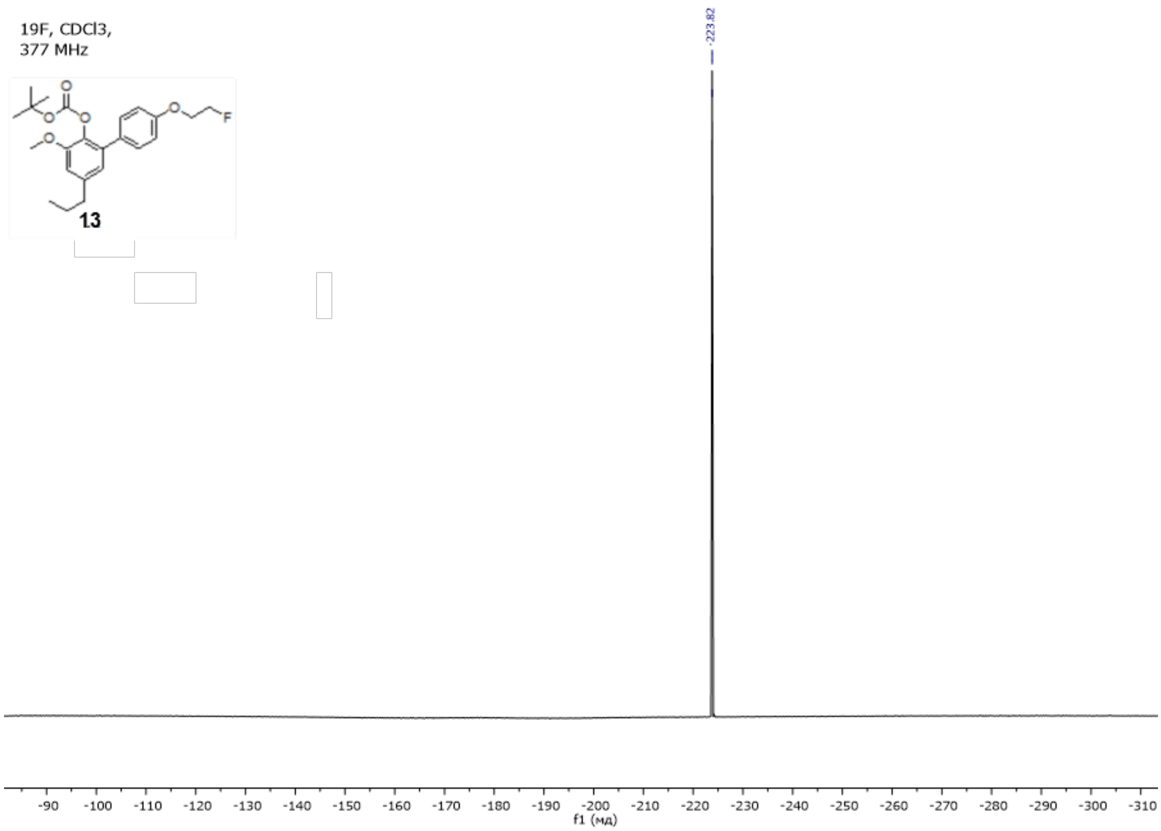

Figure S27. ^{19}F NMR spectra for compound **13**.

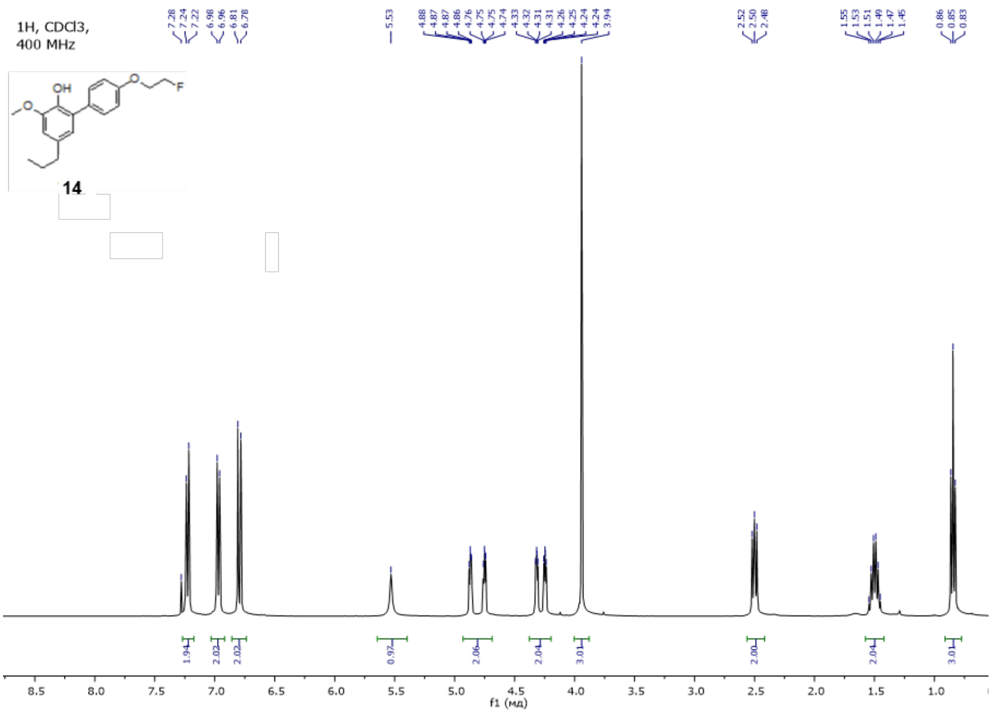

Figure S28. ^{1}H NMR spectra for compound **14**.

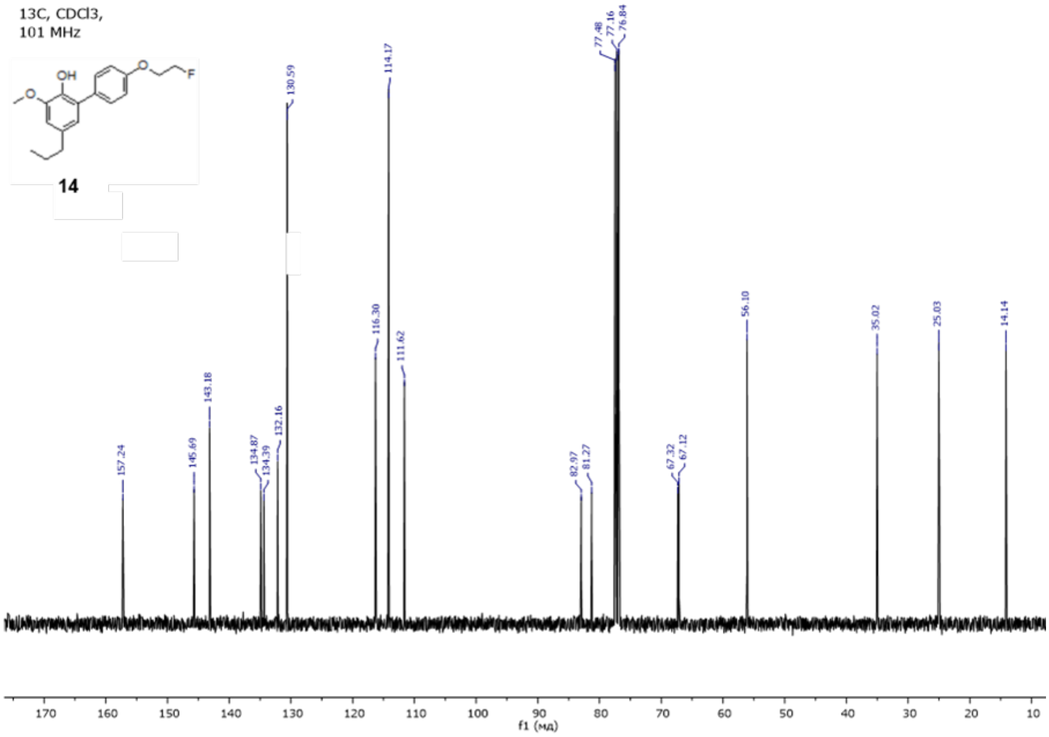

Figure S29. ^{13}C NMR spectra for compound **14**.

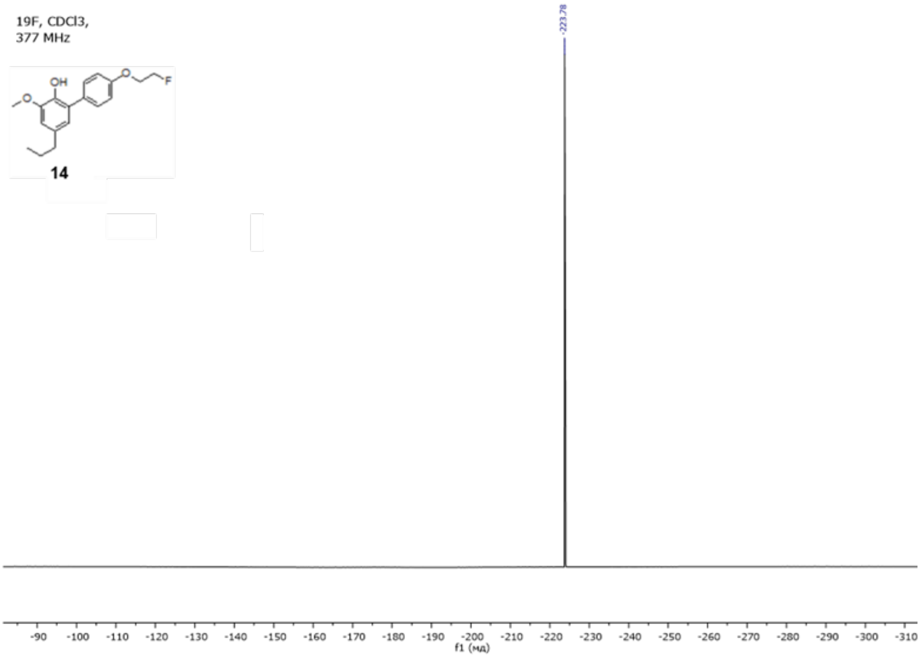

Figure S30. ^{19}F NMR spectra for compound **14**.

^{1}H NMR (400 MHz, $CDCl_3$)

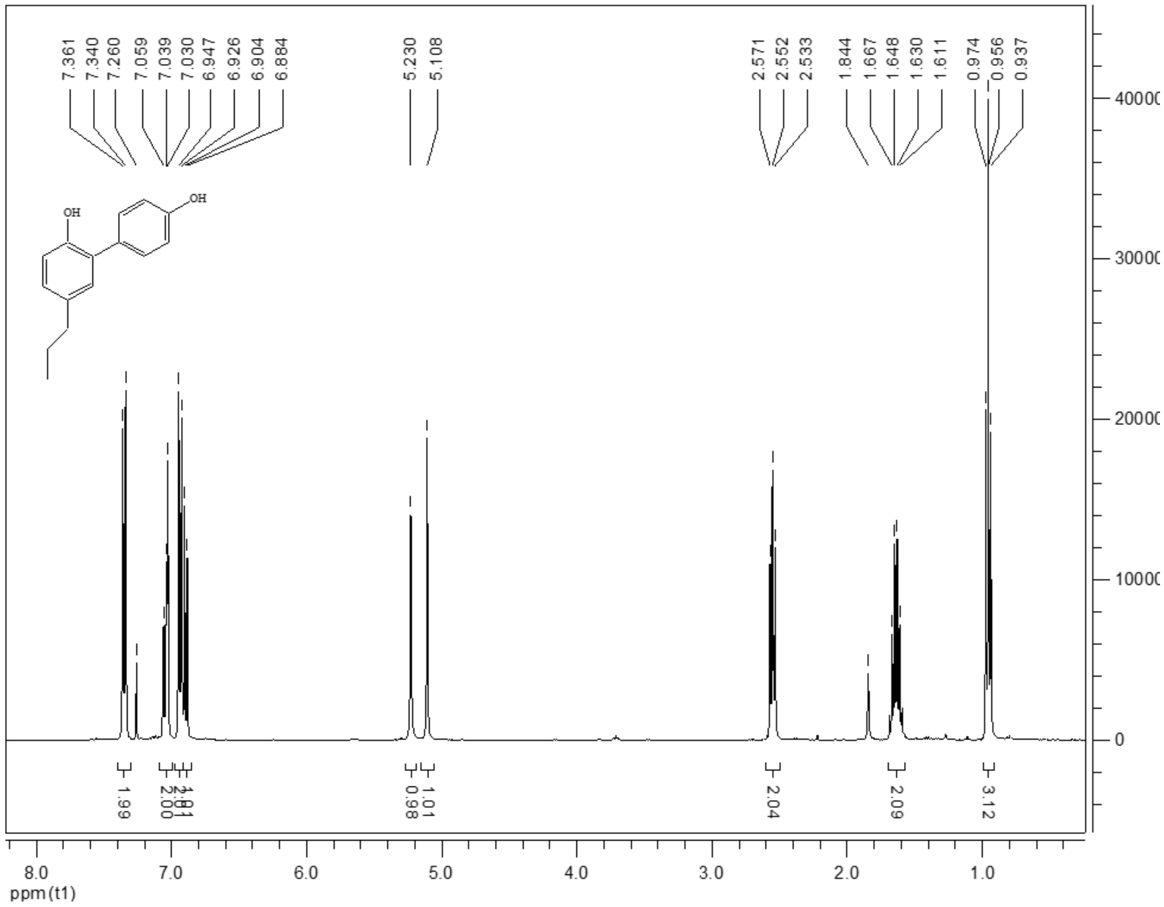

Figure S31. ^{1}H NMR spectra for **5-propyl-1,1'-biphenyl-2,4'-diol**

^{13}C NMR (101 MHz, $CDCl_3$)

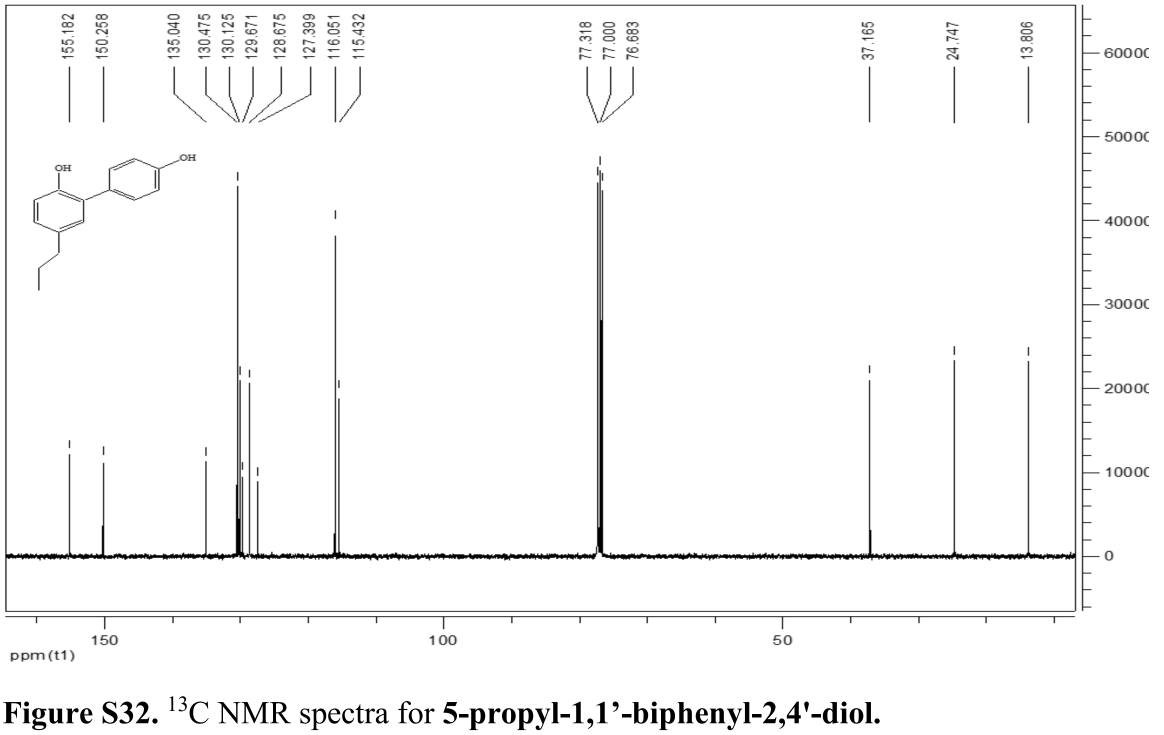

Figure S32. ^{13}C NMR spectra for **5-propyl-1,1'-biphenyl-2,4'-diol.**

II. Computational details

We computed the absolute free energies of binding for compounds MPbP, F-I, F-II, F-III and F-IV which form a congeneric set with 4'-*O*-methylhonokiol (MH) or F-IV from the equation (1)

$dG_i = dG_{MH(F\text{-}IV} + ddG_i$, (1)

where ddG_i is the relative binding free energy and $dG_{4MH(F\text{-}IV)}$ is absolute binding free energy of MH or F-IV.

The absolute free energies of binding for the new MH derivatives were computed from the equations (2, 3)

$dG = RT\ln K_b$ (2)

$dG \approx RT\ln (IC_{50})$ (3)

MH binding constant for CB-2 was known from the literature [26] as well as for MPbP in case of COX-2 [14], and for F-IV reported in this paper.

The ddG calculations were carried out using FEP+ module (Desmond 6.2) of Schrodinger 2020-2 software package. We took the agonist-bound CB-2 structure, 6PT0 from PDB data bank, since it was shown that MH and its derivatives are CB-2 receptor agonists. According to the binding assay conditions only CB-2 structure was retained from 6PT0 hetero-multimer and biologically irrelevant fragments were also removed from 6PT0 followed by preparation using Protein Preparation Wizard of Schrodinger 2020-2. The termini were capped, bond orders were assigned, hydrogens were added and missing amino acid side chains were filled with Prime module. The titratable residues protonation states were visually inspected and the complex underwent minimization (RMSD=0.5A) using OPLS3e forcefield which was used for all the simulations in this study. Similarly, COX-2 structure was also obtained as 5KIR entry from PDB data bank. The COX-2 protein were treated before simulations in the same manner. All the ligands were prepared using LigPrep and the missing torsion parameters were fitted with Force Field Builder.

For the alchemical ddG calculations to correlate well with the experiment and have good predicting power, the information of correct binding mode for the congeneric ligand series is necessary. The standard precision (SP) Glide docking was used for MH placement with post-docking minimization followed by MM-GBSA (molecular mechanics combined with generalized Born surface solvation) rescoring with only ligand minimization in a static receptor. We chose poses with the best MM-GBSA binding free energies as well as simulations described further.

For the periodic boundary condition simulations the complexes were neutralized with Cl- ions, CB-2 structures were embedded in phosphatidylcholine (POPC) lipid pre-aligned membrane according to the Orientations of Proteins in Membranes (OPM) database (https://opm.phar.umich.edu/) and placed into 20x20x15 Å SPC water box. The system was minimized by 500 ps of Brownian dynamics keeping the protein and ligand heavy atoms restrained (10 kcal/A^2*mol) and then relaxed using the default membrane relaxation protocol with end temperature being 303 K as in the binding assays. To maintain the pressure and temperature Martyna-Tobias-Klein barostat (1ps coupling) with semi-isotropic pressure scaling and Nose-Hoover thermostat were used. The further staged relaxation was carried out at 303 K in NPγT ensemble and included 5ns simulation with protein heavy atoms restrained (10 kcal/A^2*mol), then 1ns keeping protein backbone atoms restrained (5 kcal/A^2*mol) followed by 100ns simulation of unrestrained protein to let it adjust the shape for a new smaller ligand. For COX-2 the simulations end temperature 310 K was taken according to the binding assay conditions as well as isotropic pressure scaling. For each complex the MM-GBSA dGs were computed from trajectory using *thermal_mmgbsa.py* script. The free energies of binding were computed for every 100th frame and mean values were obtained in each case.

As the criteria for choosing the binding mode among the other possible ones we considered ligand RMSD values, binding site metadynamics where ligands RMSD was taken as a collective variable (CV), average dG(MM-GBSA) energy throughout the simulation. For

COX-2 we checked if the experimental ddG was accurately reproduced for a particular binding mode.

As the receptor structures for CB-2 FEP+ calculations we took trajectory frames at 50ns of simulation because they corresponded to the equilibrated systems and had protein-ligand contacts consistent with the overall picture observed during the 300ns run. Analogously, 75ns and 100ns frames for COX-2 were chosen.

For complex with chosen binding mode the ligands were aligned to MH (by maximum common substructure) for CB-2 and to F-IV for COX-2 before FEP+ calculation because accurate ddG values for these ligands have been reported. Some ligand bonds have been rotated to minimize the steric clashes with neighboring protein side chains and perturbation maps with optimal topology have been constructed. For better phase space coverage we added hypothetical intermediate 4'-(2-ethoxy)-3'-methoxy-5-propyl-2-hydroxy-1,1'-biphenyl, an analogue of F-III having hydrogen instead of fluorine, (faded salmon, thick tube in Figure 4, D) to the perturbation map in case of CB-2 complexes. Every perturbation for CB-2 consisted of 12 lambda windows 10ns long each. In case of COX-2 we took 20ns for every lambda windows to sample the configuration space more efficiently since more contacts with binding site amino acid residues were observed in this case. To enhance sampling for mutated atoms and water molecules in the binding site we used replica exchange with solute tempering (REST) and grand canonical Monte Carlo (GCMC) methods, respectively. Side chains of residues F106, I110 (CB-2) were also included in the REST "hot" atoms region for better accommodation of 2-fluoroethoxy substituent. In case of cyclooxygenase, side chains of V523 and L531 (COX-2, chain A and B, respectively) were within REST region.

For every edge the ddG values with cycle closure correction (CCC) were given. The relative binding free energies were computed at 300 K. Although the binding constant and IC_{50} were measured at 303 K (CB-2) and 310 K (COX-2) we made an assumption that for such temperature differences the binding constant is temperature-independent. Also the resulting error wouldn't exceed FEP+ method deviation.
